# Relative contributions of egg-associated and substrate-associated microorganisms to black soldier fly larval performance and microbiota

**DOI:** 10.1093/femsec/fiab054

**Published:** 2021-03-30

**Authors:** Stijn J. J. Schreven, Hugo de Vries, Gerben D. A. Hermes, Hauke Smidt, Marcel Dicke, Joop J. A. van Loon

**Affiliations:** Laboratory of Entomology, Plant Sciences Group, Wageningen University & Research, PO Box 16, 6700 AA Wageningen, The Netherlands; Laboratory of Microbiology, Agrotechnology & Food Sciences Group, Wageningen University & Research, PO Box 8033, 6700 EH Wageningen, The Netherlands; Laboratory of Microbiology, Agrotechnology & Food Sciences Group, Wageningen University & Research, PO Box 8033, 6700 EH Wageningen, The Netherlands; Laboratory of Microbiology, Agrotechnology & Food Sciences Group, Wageningen University & Research, PO Box 8033, 6700 EH Wageningen, The Netherlands; Laboratory of Entomology, Plant Sciences Group, Wageningen University & Research, PO Box 16, 6700 AA Wageningen, The Netherlands; Laboratory of Entomology, Plant Sciences Group, Wageningen University & Research, PO Box 16, 6700 AA Wageningen, The Netherlands

**Keywords:** 16S rRNA gene, amplicon sequencing, qPCR, sterile, *Hermetia illucens*, chicken manure

## Abstract

Larvae of the black soldier fly (BSF) can be used to convert organic waste into insect biomass for animal feed. In this process, they interact with microorganisms originating from the substrate, the insect and the environment. The substrate is the main determinant of the larval gut microbiota composition, but inoculation of the substrate with egg-associated bacteria can improve larval performance. We aimed to quantify the relative importance of substrate-associated and egg-associated microorganisms in BSF larval performance, bacterial abundance and bacterial community composition, when larvae were fed with chicken feed or chicken manure. For this, we inactivated substrate-associated microorganisms by autoclaving, or disinfected BSF eggs. Larval survival, weight and proportion of prepupae were determined on day 15. We collected substrate and larval samples on days 0 and 15 and performed 16S rRNA gene-targeted qPCR and amplicon sequencing. In both chicken feed and chicken manure, egg disinfection did not cause any difference in larval performance or overall microbiota composition. In contrast, in chicken manure, substrate-associated microorganisms increased larval biomass and sterilizing the substrate caused major shifts in microbiota. Thus, substrate-associated microorganisms impact not only larval microbiota but also larval performance, whereas egg-associated microorganisms have a minor role in the densities present.

## Teaser

Microbes from the feed substrate have a large impact on black soldier fly larval microbiota and performance, whereas microbes from the insect eggs only play a minor role.

## INTRODUCTION

The saprophagous larvae of the black soldier fly [BSF, *Hermetia illucens* (Linnaeus 1758); Diptera: Stratiomyidae] can be used to convert organic waste streams into insect biomass for livestock feed (Cickova *et al*. 2015; Pastor *et al*. 2015; Barragán-Fonseca, Dicke and Van Loon 2017; Wang and Shelomi 2017). These fly larvae interact with a community of microorganisms such as bacteria and fungi during the consumption of decaying organic matter (De Smet *et al*. 2018; Gold *et al*. 2018). The microbial decomposers can originate from the organic waste substrate, the insect or the environment (Benbow *et al*. 2019). Especially in nutrient-rich substrates, competition can be fierce and favours those who can monopolize the resource or exploit it fastest (Hanski 1987). The strong competition may also favour partnerships between insect hosts and their associated microbiome (Benbow *et al*. 2019). For example, bacteria on housefly eggs can suppress fungal competitors of larval offspring in manure, and *Drosophila* adults and larvae regulate yeast density and composition in rotting fruits to favour yeasts palatable to larvae and most attractive to ovipositing flies (Lam *et al*. 2009; Stamps *et al*. 2012; Buser *et al*. 2014).

The BSF larval gut bacterial community consists of a combination of ingested substrate bacteria and bacteria that are found mainly in the larvae and may originate from the eggs (Zheng *et al*. 2013; Bruno *et al*. 2019; Jiang *et al*. 2019; Wynants *et al*. 2019; Schreven *et al*. submitted). In general, the feed substrate is the main determinant of the larval gut bacterial community (Jeon *et al*. 2011; Bruno *et al*. 2019; Jiang *et al*. 2019; Zhan *et al*. 2020; Schreven *et al*. submitted). Larval and substrate bacterial communities can differ in composition depending on the feed substrate due to the flexible digestive system of the BSF larvae (Bonelli *et al*. 2020; Zhan *et al*. 2020; Schreven *et al*. submitted). Over time, the larvae alter substrate bacterial community composition by inhibiting certain bacteria while dispersing gut bacteria into the substrate (Lalander *et al*. 2013; Gold *et al*. 2018; Vogel *et al*. 2018; Jiang *et al*. 2019; Wynants *et al*. 2019; Schreven *et al*. submitted).

In other saprophagous fly species, bacteria serve directly as a larval food source (Thompson *et al*. 2013), and BSF is likely no exception. Ingested bacteria are lysed in the middle midgut by the low luminal pH and a high lysozyme activity, and the released nutrients can be absorbed in the posterior midgut (Gold *et al*. 2018; Bonelli *et al*. 2019). Additionally, bacteria can complement the digestive capabilities of an insect host (Engel and Moran 2013). BSF egg-associated and larval gut-associated bacteria can possess specific enzymes that break down macronutrients and recalcitrant macromolecules; for example, a strain of *Morganella morganii* isolated from larvae fed pig feed exhibited cellulase activity (Yu *et al*. 2010; Kim *et al*. 2014; Lee *et al*. 2014).

BSF larval performance can increase when the feed substrate is inoculated with single strains or mixtures of bacteria. When fed chicken manure inoculated with larval gut-associated strains of *Bacillus subtilis*, BSF larvae grew larger and developed faster, and conversion efficiency and adult size increased (Yu *et al*. 2011; Xiao *et al*. 2018; Mazza *et al*. 2020). In a similar set-up, egg-associated *Lysinibacillus boronitolerans*, *Kocuria marina* or *Proteus mirabilis* inoculated into chicken manure produced larger larvae and reduced the manure residue (Mazza *et al*. 2020). Mixtures of these three bacteria and *B. subtilis* also increased larval weight, fat content and protein content, depending on the ratio of strains in the mixture (Mazza *et al*. 2020). However, some bacterial strains and mixture ratios had no effect or even an adverse effect (Mazza *et al*. 2020). Commercially available bacterial mixtures, probiotics (e.g.*Lactobacillus buchneri*) and egg-associated bacteria (e.g.*Klebsiella oxytoca*) from other fly species can also improve BSF larval performance and alter BSF nutrient composition (Zheng *et al*. 2012; Skaro 2018; Somroo *et al*. 2019). The above studies on the effects of bacteria on larval performance have focused on egg-associated or larva-associated bacteria and commercially available probiotics. However, the substrate has a large effect on larval gut bacterial community composition, so the effects of substrate-associated bacteria on larval performance are potentially much larger. Moreover, the effects of single bacterial strains or mixtures of several species on BSF performance when added to a non-sterile substrate may differ from their effects in the concentration and assemblage of dozens to hundreds of bacterial species as present in the substrate or larval gut (Jeon *et al*. 2011; Jiang *et al*. 2019; Wynants *et al*. 2019; Klammsteiner *et al*. 2020; Schreven *et al*. submitted).

In this study, we aimed to quantify the relative importance of microorganisms originating from substrate or eggs in BSF larval performance and in shaping the larval and substrate microbiota, focusing on the bacterial community. We investigated this in chicken feed and chicken manure. We experimentally heat inactivated substrate-associated microorganisms and eliminated egg-associated microorganisms, and then tested for differences in larval performance parameters (survival, weight, proportion of prepupae), bacterial abundance and community composition. Because BSF larvae are used in industrial-scale bioconversion of organic waste into animal feed products (Barragán-Fonseca, Dicke and Van Loon 2017), understanding these host–microbe interactions may help improve conversion efficiency and microbiological safety of the insects as livestock feed (EFSA 2015; Bosch *et al*. 2019).

## METHODS

### Insects

Eggs were collected in corrugated cardboard strips on a moist substrate of sawdust and larval frass, from the BSF colony of the Laboratory of Entomology, Wageningen University & Research. The colony has been established with source material from the United States in 2008 and is maintained in a controlled climate chamber at 27 ± 1°C,  70 ± 10% relative humidity and photoperiod of 16 h light and 8 h dark. Larvae are reared on chicken feed (‘Kuikenopfokmeel 1’, Kasper Faunafood, Woerden, The Netherlands). Neonate larvae (<24 h after hatching) were used in the experiments.

### Egg disinfection

Upon collection, eggs were divided per three to four clutches in 1.5-mL tubes using a sterile cotton swab soaked in sterile phosphate-buffered saline (PBS). Next, the egg clutches were agitated in PBS–Tween (PBS with 0.05% Tween 20, Sigma-Aldrich Inc., Saint Louis, MO, USA) to deglutinate eggs by vortexing for 10 s, then separating eggs of the remaining clutches by gently pressing and rolling a cotton swab in the tube, and vortexing again for 10 s. PBS–Tween was removed by pipetting. For disinfection, we added 1 mL 70% ethanol to each 1.5-mL tube with eggs, vortexed 2× for 10 s, removed the liquid, added 1 mL 0.05% NaOCl, vortexed 2× for 10 s and again removed the liquid. Eggs were then rinsed three times with each 1 mL sterile PBS (1× vortexing for 10 s each). After the third rinse, 800 μL of the liquid was removed; the remaining 200 μL liquid and eggs were plated on sterile lysogeny broth agar (tryptone 10 g L^–1^, yeast extract 5 g L^–1^, sodium chloride 5 g L^–1^ and agar 15 g L^–1^) and incubated in a controlled climate chamber at 27 ± 1°C and 70 ± 10% relative humidity. After 72 h incubation, sterility was assessed [colony-forming units (CFU) per plate] and only the plates with no colonies were used in the experiment. For neonate collection, 2 mL sterile PBS was pipetted onto the lysogeny broth agar plate or its lid with neonate larvae. The suspension of PBS and neonate larvae was poured into an empty sterile Petri dish, photographed, counted and poured onto the substrates in containers of the experiment. From the agitation step onward, all steps were performed in a class II biological safety cabinet.

### Experimental set-up and preparation of feed substrates

**Figure 1.**
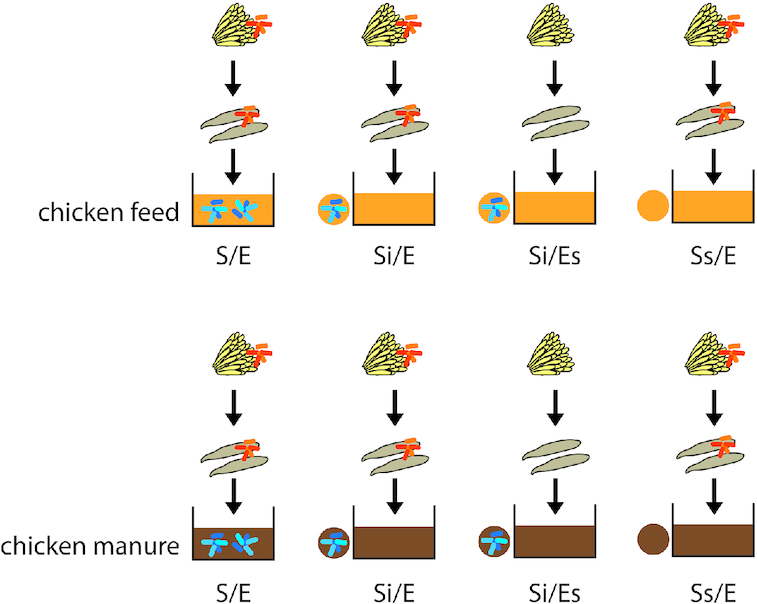
Experimental design of the study. We tested four treatments in chicken feed and chicken manure: S/E = untreated substrate with larvae from untreated eggs; Si/E = sterilized substrate with inoculum (10% w/w of untreated substrate) and larvae from untreated eggs; Si/Es = sterilized substrate with inoculum and larvae from disinfected eggs; and Ss/E = sterilized substrate without inoculum and with larvae from untreated eggs.

We tested the contribution of substrate-associated and egg-associated microorganisms using four treatments within two feed substrates (Fig. 1; Table 1). Treatment S/E was included as a control to distinguish the effect of autoclaving from the effect of microorganisms. Comparison of treatments Si/E and Si/Es will reveal the contribution of egg-associated microorganisms, whereas comparison of treatments Si/E and Ss/E will reveal the contribution of substrate-associated microorganisms. Chicken feed and fresh chicken manure were used as the two feed substrates. Chicken feed was the same as used for maintaining the BSF colony. The chicken feed was sieved (mesh size 1.5–2 mm), after which 2 mL of autoclaved demineralized water (Milli-Q^®^, Merck KGaA, Darmstadt, Germany) was added per gram dry matter (DM) of chicken feed. Part was autoclaved at 140°C for 3 h (sterilized substrate) at Unifarm, Wageningen University & Research, stored at 4°C before and after, and picked up 1 day later; simultaneously, the other part was stored in the fridge at 4°C for the same duration, i.e. 2 days (untreated substrate). Because of practical issues, we had to resort to a large-scale autoclave (volume 6500 L, Maschinenbau Scholz GmbH & Co. KG, Coesfeld, Germany) where the high temperature (140°C) and long exposure duration (3 h) were required to guarantee heat penetration for substrate sterilization. Fresh chicken manure was collected from a local organic poultry farm on the morning of substrate preparation. Without the addition of water, untreated and sterilized manure substrates were stored and prepared as described above for chicken feed. We determined DM content of each batch (both untreated and sterilized) of both chicken feed and manure in triplicate by oven-drying subsamples at 70°C for 1 day.

**Table 1.** Definition of the experimental treatments.

Treatment code	Definition	Amount of untreated substrate (g dry matter)	Amount of sterilized substrate (g dry matter)
S/E	Control treatment, comprising untreated substrate and larvae from untreated eggs	20	0
Si/E	Sterilized substrate with 10% untreated substrate as inoculum (i) and larvae from untreated eggs	2	18
Si/Es	Sterilized substrate with 10% untreated substrate as inoculum and larvae from sterilized (s; surface-disinfected) eggs	2	18
Ss/E	Sterilized substrate (s; without inoculum) and larvae from untreated eggs	0	20

Each treatment was replicated in transparent polypropylene Microbox containers O95/114+OD95/114, volume 520 mL, with a ‘#40 green filter’ in the lid to allow sufficient air exchange but prevent microbial contamination (SacO_2_, Deinze, Belgium; autoclaved before use). Per container, a total of 20 g DM feed substrate was used, composed of untreated and/or sterilized substrate (Table   1). All feed was provided at the start of the experiment because later replenishment would disturb the effects of our treatments on microbial community dynamics. Based on the DM content, sterile Milli-Q water was added to the substrate in the container, in order to obtain a DM content of 33% in all substrates. To control for loss of vitamins during autoclaving, we added 1 mL of 0.35 g mL^–1^ stock solution of Vanderzant vitamin mixture for insects (Sigma-Aldrich Inc., Saint Louis, MO, USA) to the substrate in each container—also to untreated substates to ensure all substrates contain at least this amount of vitamins. After these additions, the substrate in each container was mixed thoroughly using a sterile spatula. The distribution of the substrate into containers, addition of water and vitamins, and mixing were done in a class II biological safety cabinet. Containers with substrate were then incubated at 27 ± 1°C and 70 ± 10% relative humidity until the next day (start of experiment, day 0), especially to allow microbial populations to establish in the inoculated treatments. Although we aimed to standardize the amount of feed substrate and moisture content, treatments differed in these parameters in both chicken feed and chicken manure (Table S1, Supporting Information).

After the incubation of substrates, 100 neonate larvae were added to each container in the class II biological safety cabinet. Each treatment of chicken feed was replicated four times, divided over two batches. Each treatment of chicken manure was replicated six times, divided over three batches. The third batch of manure was included because the second batch had larvae of almost 24 h since hatching. Finally, the containers were placed in a controlled climate chamber of 27 ± 1°C,  70 ± 10% relative humidity and photoperiod of 16 h light and 8 h dark. Within each batch, containers were repositioned randomly each day to account for any temperature or humidity gradients in the climate chamber. Fifteen days after the addition of the larvae to the substrate, the batch was harvested. Only the treatment of autoclaved chicken feed (Ss/E) was continued until day 22 to increase the chance of successful DNA isolation, because larvae were very small on day 15.

### Sampling for molecular analyses of microbial composition

Samples for DNA extraction were collected from eggs, larvae and substrates to assess sterility of disinfected eggs and autoclaved substrates, and to compare bacterial communities of substrates and eggs/larvae at the start and end of the experiment. Untreated eggs were transferred directly per three to four clutches from the cardboard strip into a 2-mL tube, using a sterile cotton swab soaked in sterile PBS. Disinfected eggs and the 200 μL remaining liquid of the third rinse PBS were transferred by pipet to a 2-mL tube. The egg samples were collected on the day of egg collection and disinfection, i.e. 3 days before adding the neonate larvae to the substrates. Substrate samples were collected on day 0 (onset of experiment, i.e. day that neonate larvae were added to the substrate) and day 15, prior to larval sampling, using a sterilized plastic straw to take a vertical core from the substrate. In cases where this was unsuccessful, a sterile spatula was used. Larval samples of day 15 were collected by picking three average-sized larvae of a container using sterile tweezers (or six larvae in chicken feed Ss/E, since larvae were 3–5 mm length instead of 15–25 mm). Larvae were then surface-disinfected using ethanol and bleach according to the following protocol: 30 s sterile Milli-Q water, 30 s 70% ethanol, 30 s 1% Halamid^®^-D (chloramine-T, Veip Disinfectants, Wijk bij Duurstede, The Netherlands) and 2 × 10 s in sterile Milli-Q water. Each rinsing step was done in a separate 65-mm Petri dish. Sampling was done in a class II biological safety cabinet and all samples for molecular analyses were snap-frozen in liquid nitrogen.

### pH measurements

On days 0 and 15, additional substrate samples of 1–2 g were collected from each container for pH measurement. These samples were stored at −20°C. pH was measured after thawing and suspending 1 g of each sample in 10 mL Milli-Q water, using a pH meter (ProLine B210, ProSense B.V., Oosterhout, The Netherlands).

### Larval performance

After sampling for molecular analyses, the content of the container was harvested outside the biological safety cabinet. Larvae were separated from residue, washed in a sieve under lukewarm tap water, dried with tissue and counted. Larval biomass samples were then stored at −20°C. DM content of the residue was determined by weighing a fresh residue sample and drying it in an oven at 70°C until stable weight. Additionally, fresh samples of each residue were stored at −20°C. Subsamples of 10 average-sized larvae of each frozen sample were also weighed and oven-dried at 70°C until stable weight, to determine DM content and individual larval weight (g DM). Total larval biomass (g DM) was calculated as the individual larval weight (g DM) multiplied by the total number of surviving larvae on the day of harvest.

### Processing of samples for molecular analyses

Samples were ground in liquid nitrogen using disinfected mortar and pestle. Approximately 50 mg of sample was then weighed (to 0.001 g precision) and transferred to a 1.5-mL Eppendorf tube. Samples were randomly processed in batches of 16 samples, using the method of cell lysis, repeated bead-beating and DNA extraction adapted from Salonen *et al*. (2010) and Van Lingen *et al*. (2017). Per 70 samples, two no-template controls (NTCs) were included to control for DNA isolation kit contaminants (isolation blank). Three hundred microlitres buffer for Stool Transport and Recovery (STAR, Roche Molecular Systems Inc., Pleasanton, CA, USA) was added to the tube and vortexed until all frozen sample was suspended (10–20 s). The suspension was transferred to a sterile 2.0-mL screw-cap tube containing 0.1 g zirconia beads and three glass beads of 2.5 mm diameter. The samples were then homogenized in a bead beater (Precellys 24, Bertin Technologies, Montigny-le-Bretonneux, France) for 3 × 1 min at 5.5 m s^–1^ with a waiting step of 20 s in between, followed by incubation for 15 min at 95°C and 300 rpm, and centrifugation for 5 min at 16 100 × *g* and 4°C. Supernatant was transferred to a new tube. The homogenization, incubation and centrifugation were repeated with fresh 200 μL STAR buffer, and the supernatant was combined with the first supernatant. DNA was then isolated from 250 μL pooled supernatant by adding it to a cartridge of the Maxwell 16 Tissue LEV Total RNA Purification Kit (cat. no. XAS1220; Promega Corporation, Madison, WI, USA) and eluted in 30 μL nuclease-free water using the Maxwell MDx robot (Promega Corporation). DNA concentration was measured using a Qubit dsDNA Broad Range Assay Kit (Thermo Fisher Scientific, Waltham, MA, USA), after which samples with a DNA concentration above 50 ng μL^–1^ were diluted to 20 ng μL^–1^ for barcoded PCR.

### Quantitative PCR

Absolute quantification of bacteria was carried out using qPCR targeting the 16S rRNA gene. Extracted and purified DNA template was diluted 1:5–1:125 depending on pilot runs of qPCR with dilution series. We used the universal primers BACT1369F (5′-CGGTGAATACGTTCYCGG-3′) and PROK1492R (5′-GGWTACCTTGTTACGACTT-3′) (Van Lingen *et al*. 2017). Per reaction, a mix of 10 μL BioLine SensiFAST SYBR (Bioline Meridian Bioscience, London, UK), 1 μL 10 μM forward primer, 1 μL 10 μM reverse primer, 3 μL nuclease-free water and 5 μL (diluted) DNA template was added. qPCR was performed in a Bio-Rad CFX96 C1000 real-time PCR machine (Bio-Rad Laboratories Inc., Hercules, CA, USA), as follows: initial denaturation at 95°C for 3 min, then 40 cycles of denaturation at 95°C (10 s), annealing at 60°C (10 s) and elongation at 72°C (30 s), followed by a melt curve analysis from 65°C to 95°C in 0.5°C increments for 5 s each. All reactions were run in duplicate. Each 96-well plate contained a dilution series of a standard (and inter-run calibrator) of 2.38 × 10^3^ to 1.49 × 10^6^ 16S rRNA gene amplicons of *Bacillus circulans*, in five steps of 1:5 dilutions. Besides, each plate contained five NTCs, two of which used 5 μL of nuclease-free water from the dilutions and three used 5 μL nuclease-free water from the master mix.

Amplification curves and melting curves were checked in the Bio-Rad CFX Manager. Sample quality assessment, run efficiency, inter-run calibration and calculation of copy numbers were done using qbase+ (Hellemans *et al*. 2007; Biogazelle, Zwijnaarde, Belgium). PCR efficiency ranged between 74.1% and 90.9% for the five 96-well plates. The standard curves were used for inter-run calibration, and only the standard curve of the first run (highest efficiency, 90.9%) was used to calculate copy numbers of all samples. NTCs showed *C*_q_ values between 36.1 and 39.5. Samples within five-cycle difference of the NTC with lowest *C*_q_ value in that plate were scored as negative (22 samples and 8 DNA isolation blanks; Hellemans *et al*. 2007). Additionally, 11 out of 20 egg samples scored negative and 1 of these samples was excluded from analysis because its melting curve indicated low sample quality. Calibrated quantities of duplicates were averaged, and these averages were used to calculate the number of 16S rRNA gene copies per gram fresh matter of starting material, which were subsequently log_10_ transformed. Samples that scored negative were imputed as log_10_(1) = 0, meaning that the value was below detection threshold.

### Barcoded PCR

Bacterial community composition of samples was determined using Illumina HiSeq sequencing of amplicons of the V5–V6 region of the 16S rRNA gene. We performed barcoded PCR on samples in duplicate, with barcoded primers F784–1064R (Ramiro-Garcia *et al*. 2016). Per PCR run, we included one NTC (1 μL nuclease-free water as template) as a negative control. As positive controls, we used synthetic mock communities of known composition (Ramiro-Garcia *et al*. 2016). The below procedure is largely the same as in Schreven *et al*. submitted. For each reaction, the following 50 μL mix was prepared in duplicate: 36.5 μL nuclease-free water, 10 μL 5× Phusion HF buffer (Thermo Fisher Scientific, Waltham, MA, USA), 1 μL dNTPs (10 mM), 0.5 μL Phusion Hot Start II DNA polymerase (2U μL^–1^) (Thermo Fisher Scientific), 1 μL barcoded primers (10 μM) and 1 μL DNA template. The following PCR program was used: 98°C for 30 s, 25 cycles of 98°C 10 s, 42°C 10 s, 72°C 10 s and 72°C for 7 min. PCR products were checked for yield and correct size by agarose gel electrophoresis. Duplicate reaction products were pooled and amplified DNA was purified using the CleanPCR magnetic bead suspension (CleanNA, Waddinxveen, The Netherlands), 1.8× the volume of the PCR mix, two washes with 200 μL 70% ethanol, and eluted in 30 μL nuclease-free water. Purified DNA concentrations were measured using the Qubit dsDNA Broad Range Assay Kit and pooled in equimolar concentrations per library of 70 samples (randomly assigned to each library), concentrated using magnetic beads and re-eluted in 20 μL nuclease-free water. Final DNA concentration per sequencing library was measured in Qubit, after which the libraries were shipped to Eurofins Genomics Germany GmbH (Konstanz, Germany) for 2 × 150 bp sequencing on an Illumina NovaSeq 6000 instrument (Illumina Inc., San Diego, CA, USA).

Amplicon sequence data were processed using NG-Tax 2.0 (Poncheewin *et al*. 2019) and annotated using the SILVA 132 reference database (Quast *et al*. 2013).

### Statistical analyses

All analyses were conducted in R version 3.5.0 (R Core Team 2018).

### Larval performance

The effect of treatment on larval survival rate, percentage of prepupae, individual weight and total larval biomass was investigated per feed substrate separately. Linear mixed model (LMM) selection of a random intercept for batch effect and a variance structure for treatment was performed based on Akaike's information criterion (AIC, Sakamoto, Ishiguro and Kitagawa 1986; nlme package, Pinheiro *et al*. 2018). If the random term did not improve the model, a linear model or generalized least squares regression was used (LM or GLS, respectively). Non-parametric testing for differences between treatments was done with Kruskal–Wallis tests. Post-hoc pairwise comparisons for linear models were performed using estimated marginal means (EMM) with Tukey-corrected *P*-values (emmeans package; Lenth 2020). Non-parametric post-hoc comparisons were made using the Wilcoxon rank-sum test with *P*-values corrected for false discovery rate (FDR; Benjamini and Hochberg 1995).

### Substrate moisture content and pH

Substrate moisture content was tested for treatment effects following the same procedure as for the larval performance traits. Substrate pH was tested using a generalized linear mixed model regression (GLMM) with Gamma distribution and inverse link function since LMM residuals were not normally distributed, with a random intercept for container ID (lme4 package; Bates *et al*. 2015). Post-hoc comparisons were made using EMM with Tukey-corrected *P*-values.

### Bacterial abundance

Bacterial 16S rRNA gene abundances resulting from qPCR were tested separately per day. For substrate samples on day 0, we investigated the effect of treatment using linear model regression with AIC-based selection of a variance structure. This was done only for chicken manure, because chicken feed had insufficient replicates in other treatments than control (S/E). For larval and substrate samples of day 15, we tested the effects of treatment and sample type using an LMM regression with a random intercept for container ID, and AIC-based model selection of a variance structure. If model residuals were not normal, we performed GLMM with Gamma distribution and inverse link function. Post-hoc comparisons were made in EMM with Tukey-corrected *P*-values. Bacterial abundance of egg samples was compared between treatments using a Wilcoxon rank-sum test.

### Bacterial community composition

Sequence data were explored and analysed using the phyloseq (McMurdie and Holmes 2013) and microbiome packages (Lahti and Shetty 2017). Chloroplast and mitochondrial reads were excluded from analysis, as well as reads of contaminant amplicon sequence variants (ASVs). Contaminant ASVs were identified by visual inspection of correlation plots of relative ASV abundance against DNA concentration (ng μL^–1^) in the PCR product. Samples similar to blanks in qPCR were also excluded from analysis of sequencing data, as well as samples with fewer than 5000 reads (excluding mitochondrial, chloroplast or contaminant ASVs) since microbiota composition of these samples was considered unreliable (36 out of 140 samples). One substrate sample of autoclaved chicken manure with inoculum (Si/E) of day 0 and one substrate sample of untreated chicken manure (S/E) of day 15 were excluded from analysis because they were suspected to be erroneously mixed up in the lab workflow. Additionally, samples of two containers in the autoclaved chicken feed with untreated eggs (Ss/E) were excluded because these were heavily contaminated with a green fungus, unlikely to originate from the eggs. All subsequent analyses were performed with relative abundance data at genus level.

Alpha diversity was measured as Faith's phylogenetic diversity, which is phylogenetically weighted richness, i.e. the sum of all phylogenetic tree branch lengths in a sample (Faith 1992). For substrates samples of day 0, we performed a linear model regression to test for a treatment effect. This was done only for chicken manure, since chicken feed had insufficient replicates in other treatments than control (S/E). For larval and substrate samples of day 15, we tested for the effects of treatment and sample type using an LMM regression with a random intercept for container ID and AIC-based selection of a variance structure. Post-hoc comparisons were made using EMM with Tukey-corrected *P*-values.

Total microbiota variation was analysed per feed substrate and day separately using non-metric multidimensional scaling (NMDS) based on weighted UniFrac distances (Kruskal 1964; Luzopone and Knight 2005). The effects of treatment and sample type were quantified using distance-based redundancy analysis (dbRDA; McArdle and Anderson 2001) and statistically tested using a permutational multivariate analysis of variance (ANOVA; anova.cca function; vegan package; Oksanen *et al*. 2019).

Weighted UniFrac distance between larval and substrate microbiota composition was assessed for differences between treatments using an ANOVA and post-hoc comparisons with Tukey correction.

Per feed substrate, the most abundant and prevalent genera [present in at least 10% of samples and comprising at least 1% of reads in a sample (or 10% in chicken manure)] were displayed in heat maps of mean relative abundance. Differences in relative abundance of these genera between treatments were tested using Kruskal–Wallis tests and post-hoc Wilcoxon rank-sum tests. *P*-values were FDR-corrected.

The sequence data underlying this article are available in the European Nucleotide Archive at https://www.ebi.ac.uk/ena/browser/view/PRJEB40821 and can be accessed with the study accession number PRJEB40821. Larval performance and substrate pH data underlying this article are included in Table S9 (Supporting Information). The software code underlying this article is available in the 4TU.ResearchData repository at https://data.4tu.nl/portal and can be accessed with the DOI 10.4121/13118294.

## RESULTS

### Larval performance

**Figure 2.**
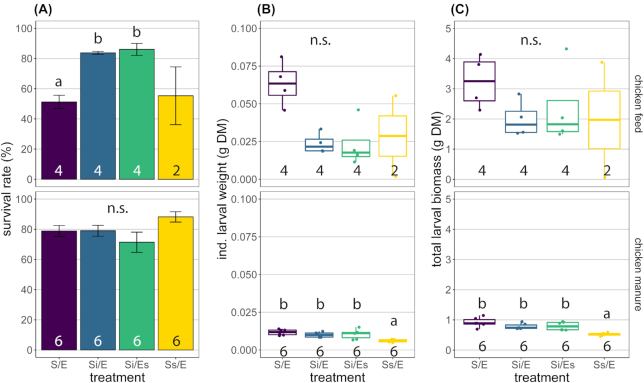
Larval performance: **(A)** survival rate (%, mean ± SE); **(B)** individual larval weight (gram dry matter, box plots); **(C)** total larval biomass (gram dry matter, box plots). Top panels are for chicken feed, bottom panels for chicken manure. Treatment codes: S/E = control treatment (untreated substrate and untreated eggs); Si/E = sterilized substrate with inoculum and untreated eggs; Si/Es = sterilized substrate with inoculum and disinfected eggs; Ss/E = sterilized substrate and untreated eggs. All treatments were harvested on day 15, except chicken feed Ss/E that was harvested on day 22 and excluded from statistics. Numbers in bars indicate sample sizes (number of containers). Means or medians with different letters are significantly different (α = 0.05, different test used per substrate and parameter). n.s. = not significant.

Larval performance was not affected by microorganisms associated with eggs in either of the feed substrates, and only by substrate-associated microorganisms in chicken manure. In chicken feed, more larvae survived in the autoclaved substrates with inoculum (Si/E, 84%; Si/Es, 86%) versus the control treatment (S/E, 51%; GLS, *P* < 0.001), but larvae tended to be heavier in the control treatment (0.063 g DM) than in the autoclaved substrates with inoculum (0.018–0.022 g DM; Kruskal–Wallis, *P* = 0.037 but no significant pairwise differences; Fig. 2A and B). No differences were observed in total larval biomass and the percentage of prepupae (Kruskal–Wallis, *P* = 0.232 and *P* = 0.070, respectively; Fig. 2C; Fig. S1, Supporting Information).

In chicken manure, larvae from autoclaved manure without inoculum were lighter (Ss/E, 0.006 g DM) compared with the other treatments (0.010–0.012 g DM; Kruskal–Wallis, *P* = 0.005; Fig. 2B). This also resulted in lower total larval biomass from this treatment (Ss/E, 0.531 g DM) compared with the other manure treatments (0.739–0.887 g DM; Kruskal–Wallis, *P* = 0.003; Fig. 2C). Survival rate and percentage of prepupae did not differ among treatments in chicken manure (ANOVA, *P* = 0.110; Kruskal–Wallis, *P* = 0.235, respectively; Fig. 2A; Fig. S1, Supporting Information).

### Substrate pH

In chicken feed, substrate pH increased from day 0 (5.6–5.7) to day 15 (7.5–8.2) in all treatments except autoclaved chicken feed without inoculum (5.5–5.6; Fig. S2 and Table S2, Supporting Information). Substrate pH of inoculated chicken feed with disinfected eggs on day 15 (Si/Es, 8.2) was higher than in inoculated chicken feed with untreated eggs (Si/E, 7.5). In chicken manure, substrate pH also increased over time (from 7.5–8.0 to 9.0–9.3) in all treatments except untreated manure (8.7–9.1; Fig. S2 and Table S3, Supporting Information). Additionally, on day 0, autoclaved manure without inoculum had a significantly lower pH (7.5) than untreated manure (8.7).

### Substrate moisture content

Substrate moisture content on day 15 differed among treatments in both chicken feed and chicken manure (Kruskal–Wallis, chicken feed: *P* = 0.021; chicken manure: *P* = 0.004; Fig. S3, Supporting Information). Untreated chicken feed was wetter (83%) than the other treatments (79–80%); and autoclaved chicken manure without inoculum was drier (64%) than the other manure treatments (67–68%).

### Total bacterial abundance

**Figure 3.**
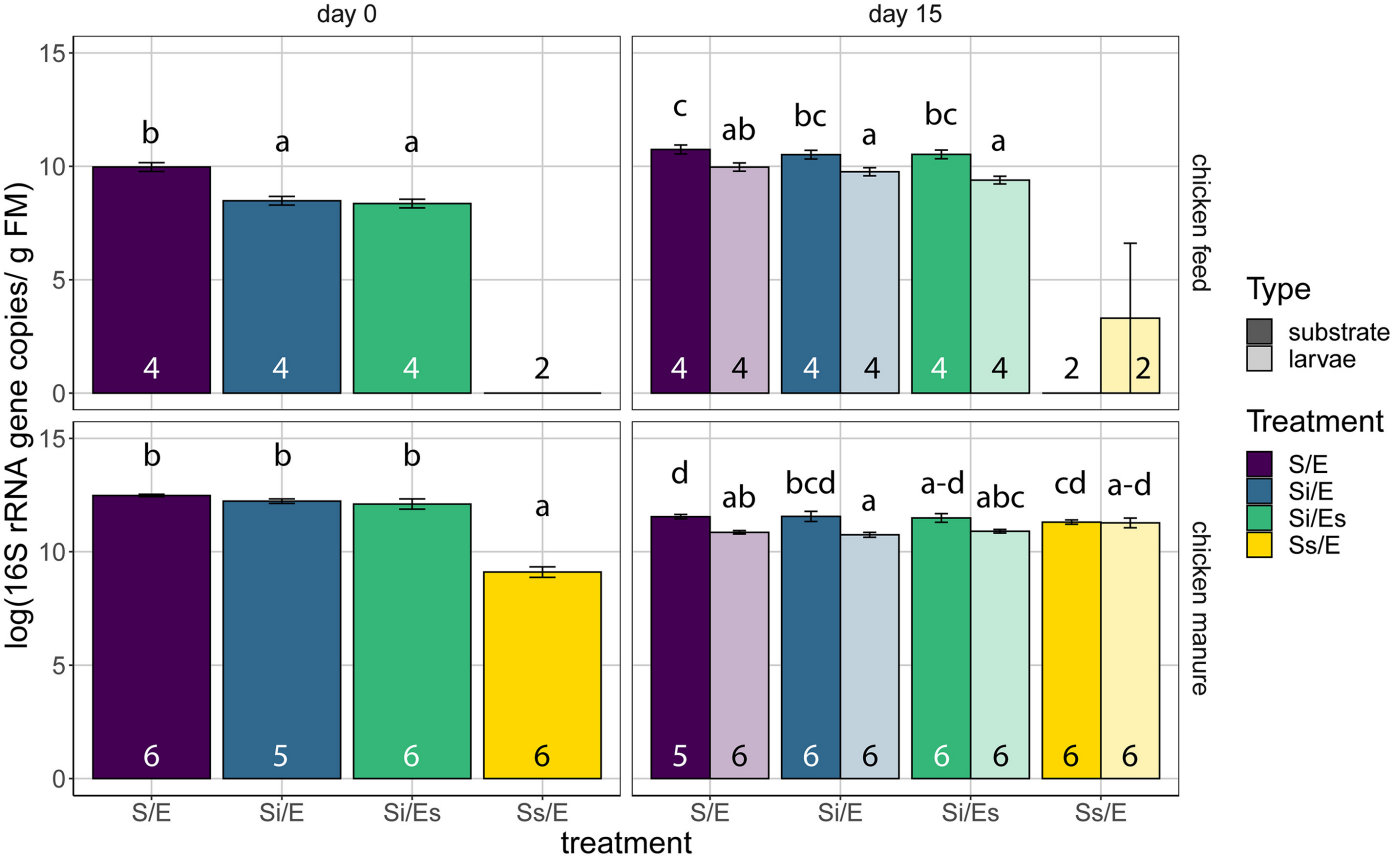
Total bacterial 16S rRNA gene abundance (estimated marginal mean ± SE, log_10_ 16S rRNA gene copies per gram fresh matter), on day 0 and 15 in substrate and larval samples in the different treatments of chicken feed (top panels) and chicken manure (bottom). Treatment codes: S/E = control treatment (untreated substrate and untreated eggs); Si/E = sterilized substrate with inoculum and untreated eggs; Si/Es = sterilized substrate with inoculum and disinfected eggs; Ss/E = sterilized substrate and untreated eggs. Numbers in bars indicate sample sizes (number of containers). Larvae were only sampled on day 15. Samples that scored similar to no-template controls were imputed in the analysis as log_10_(1) = 0, meaning that 16S rRNA gene copy numbers were below the detection threshold. Means without shared letters are significantly different (tested per feed substrate and day; α = 0.05; post-hoc comparisons with Tukey-corrected *P*-values).

Treatments in both chicken feed and chicken manure differed in bacterial 16S rRNA gene abundance on day 0 (chicken feed, LM, *P* < 0.001; chicken manure, GLS, *P* < 0.001; Fig. 3). In chicken feed, larval samples in all treatments except Ss/E contained fewer 16S rRNA gene copies than substrates (10^9^–10^10^ vs 10^11^ copies g^–1^ sample; GLMM, *P* < 0.001; Fig. 3). 16S rRNA gene abundance did not differ between chicken feed S/E, Si/E and Si/Es treatments (*P* = 0.154). All substrate samples and three larval samples of Ss/E scored as negative (*C*_q_ values within five cycles of the negative control with the lowest *C*_q_ value).

Autoclaved manure without inoculum (Ss/E) still contained considerable (10^9^) numbers of 16S rRNA gene copies per gram sample on day 0. After 15 days, no differences in bacterial 16S rRNA gene abundance were found between treatments of chicken manure (LMM, *P* = 0.020 but no significant post-hoc comparisons), but in treatments S/E and Si/E, larval samples contained fewer 16S rRNA gene copies than substrates (10^11^ vs 10^12^; *P* < 0.001).

The number of 16S rRNA copies per gram of eggs did not differ significantly between untreated and disinfected eggs (Wilcoxon rank-sum test, *P* = 0.289; Fig. S4, Supporting Information).

### Bacterial community composition

Amplicon sequencing resulted in 31 million reads assigned to 4231 ASVs [excluding chloroplast or mitochondrial reads (2.2% of all reads) and contaminant ASVs (2.4%)]. We identified 188 contaminant ASVs, which were mostly assigned to known lab contaminant genera, e.g.*Ralstonia* and *Cupriavidus*, and were filtered from our dataset (Salter *et al*. 2014; Table S4, Supporting Information). In the positive controls (synthetic mock communities), Spearman rank correlations at genus level between replicates were high: 0.89–0.99 (mean 0.95) for mock community 3 and 0.91–0.99 (mean 0.95) for mock community 4. Spearman rank correlations at genus level between positive controls and corresponding theoretical mock composition were 0.79 ± 0.04 for mock 3 and 0.73 ± 0.02 for mock 4, which is in accordance with routinely observed values, indicating accurate and reproducible sequencing of bacterial communities across sequencing runs.

DNA isolation and PCR replicates were highly correlated in both untreated chicken feed and chicken manure for substrates of day 0 and for larvae (Table S5, Supporting Information). PCR replicates of egg samples did not result in reproducible bacterial communities (untreated eggs: *r* = 0.22–0.39; disinfected eggs *r* = 0.12), and so we decided not to analyse the egg samples further.

### Alpha diversity

**Figure 4.**
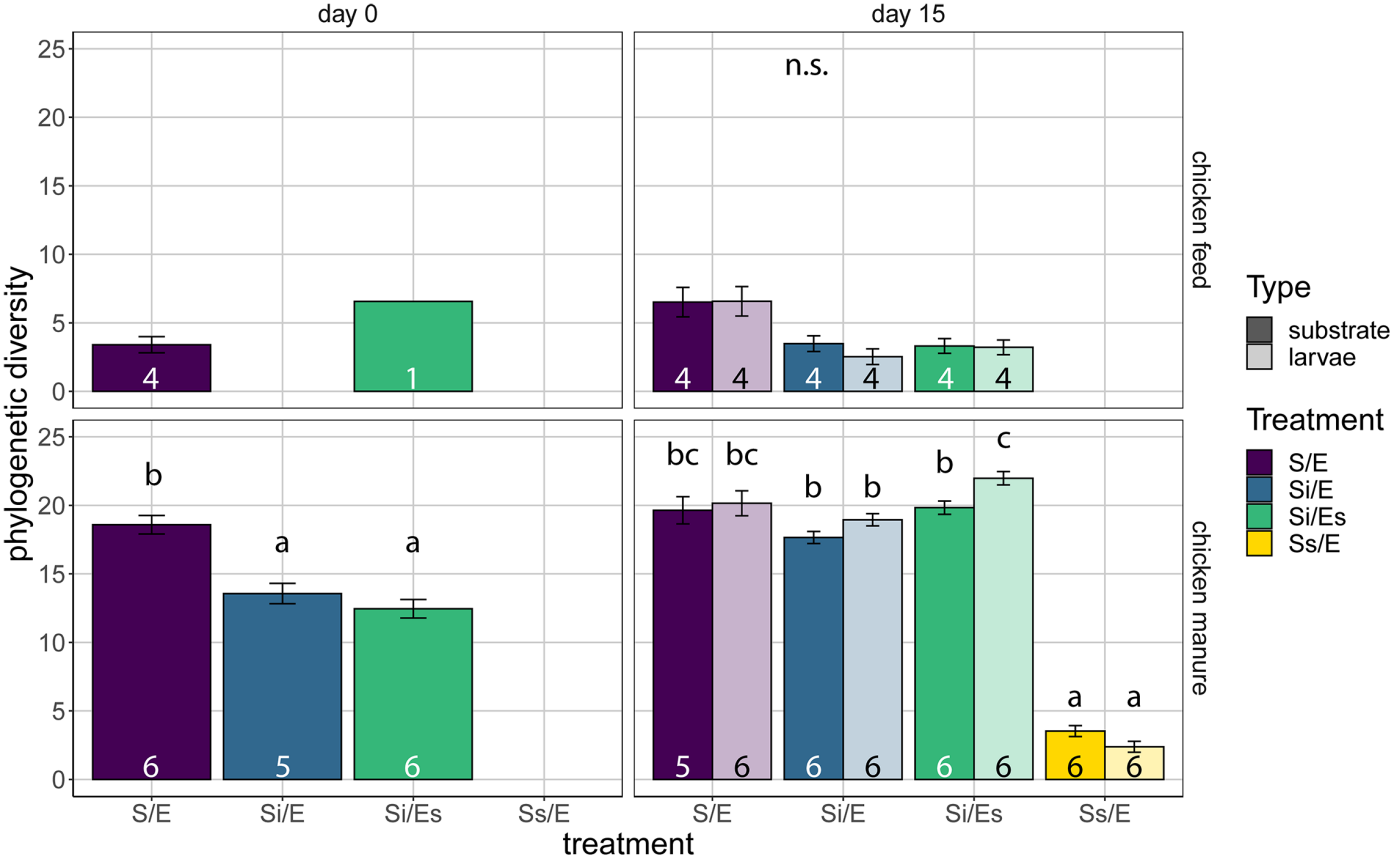
Faith's phylogenetic diversity (mean ± SE) of substrate and larvae samples of the different treatments in both feed substrates. Top panels are for chicken feed, bottom panels for chicken manure. Treatment codes: S/E = control treatment (untreated substrate and untreated eggs); Si/E = sterilized substrate with inoculum and untreated eggs; Si/Es = sterilized substrate with inoculum and disinfected eggs; Ss/E = sterilized substrate and untreated eggs. Numbers in bars indicate sample sizes (number of containers). Larvae were only sampled on day 15. Means that share no letters are significantly different (tested per substrate and day; α = 0.05; post-hoc comparisons with Tukey-corrected *P*-values).

In chicken feed on day 15, there was a significant treatment effect on Faith's phylogenetic diversity, but pairwise comparisons showed no differences (LMM, *P* = 0.015; Fig. 4). In chicken manure on day 0, untreated manure had a higher phylogenetic diversity (S/E, 18.6) than inoculated manure (Si/E, 13.6; Si/Es, 12.5) (ANOVA, *P* < 0.001; Fig. 4). Fifteen days later, substrate microbiota of untreated and inoculated manure groups did not differ (17.7–19.8), but the microbiota of autoclaved manure was less diverse than the rest (Ss/E, 3.5) (LMM, *P* < 0.001; Fig. 4). In addition, larval microbiota was more diverse than substrate microbiota in the inoculated manure with disinfected eggs (Si/Es, 22.0 vs 19.8) (*P* = 0.001; Fig. 4).

### Beta diversity

**Figure 5.**
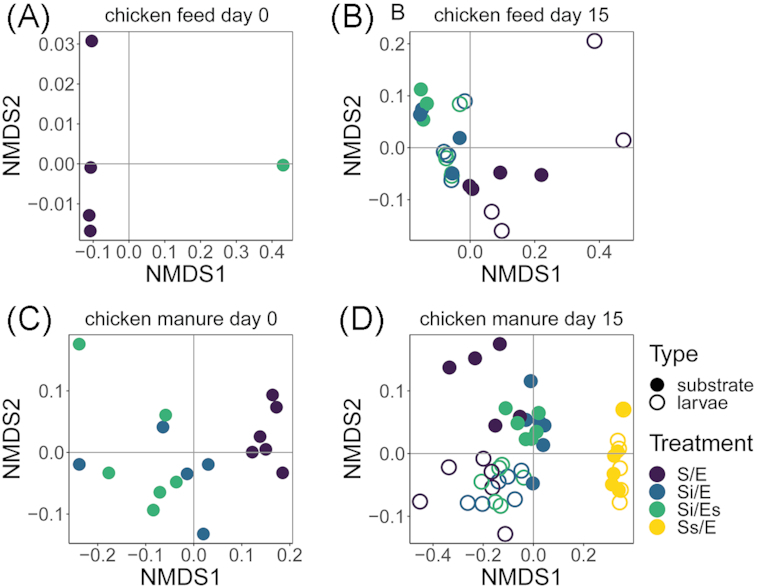
Bacterial community composition (NMDS), based on weighted UniFrac distances at genus level: **(A)** chicken feed substrates on day 0; **(B)** chicken feed larvae and substrates on day 15; **(C)** chicken manure substrates on day 0; **(D)** chicken manure larvae and substrates on day 15. Treatment codes: S/E = control treatment (untreated substrate and untreated eggs); Si/E = sterilized substrate with inoculum and untreated eggs; Si/Es = sterilized substrate with inoculum and disinfected eggs; Ss/E = sterilized substrate and untreated eggs. Stress of NMDS solutions: A = 0, B = 0.063, C = 0.066, D = 0.060.

Treatments affected microbiota composition in both chicken feed and chicken manure (weighted UniFrac NMDS and dbRDA; Fig. 5; Tables S6 and S7, Supporting Information). The most abundant phyla were Firmicutes, Proteobacteria, Actinobacteria and Bacteroidetes (Fig. S5, Supporting Information).

**Figure 6.**
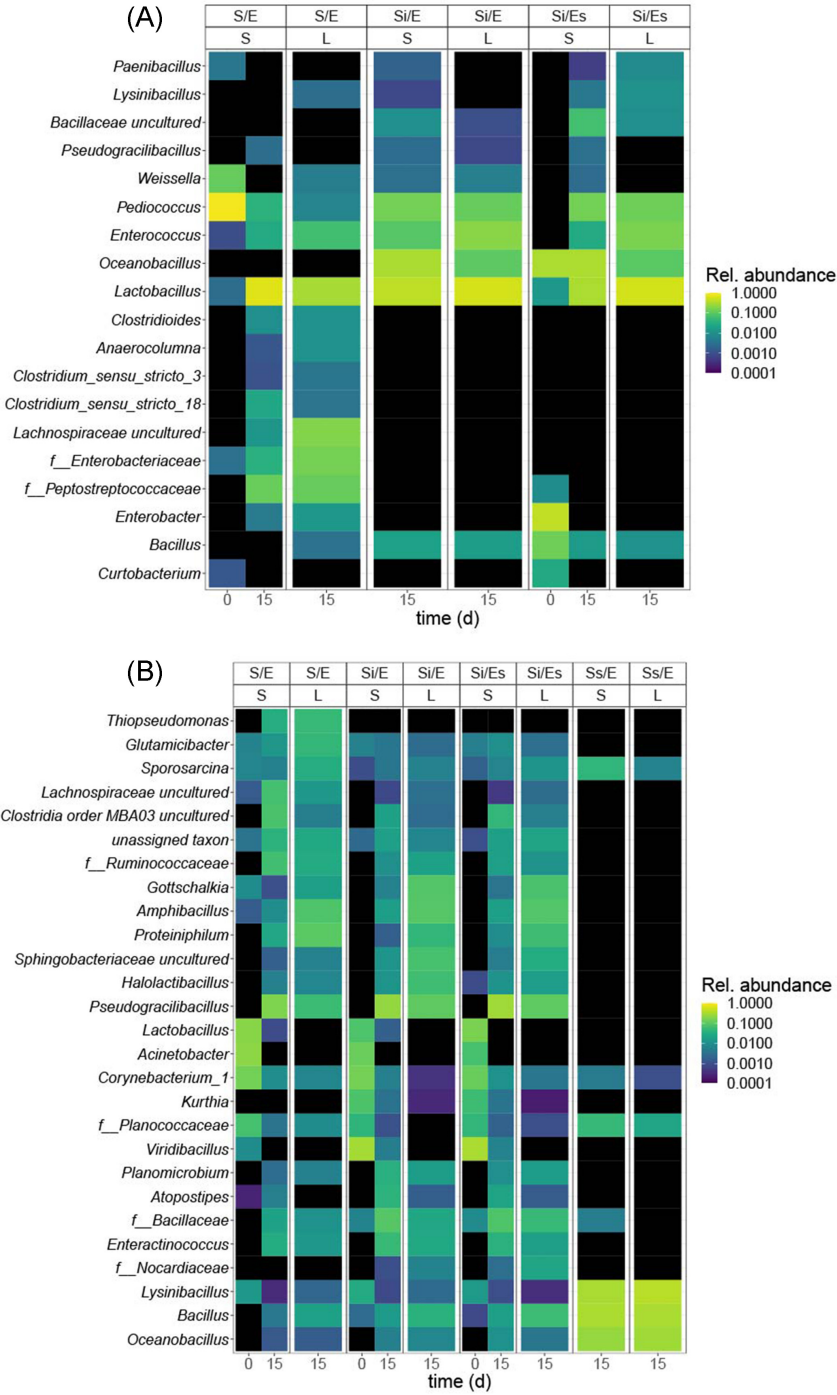
Heat maps of the most abundant genera per substrate, mean relative abundance: **(A)** chicken feed; **(B)** chicken manure. Treatment codes: S/E = control treatment (untreated substrate and untreated eggs); Si/E = sterilized substrate with inoculum and untreated eggs; Si/Es = sterilized substrate with inoculum and disinfected eggs; Ss/E = sterilized substrate and untreated eggs. S = substrate microbiota, L = larval microbiota. For chicken feed, genera are displayed if relative abundance is >1% in a sample, and occurring in >10% of all samples; for chicken manure, genera are displayed if relative abundance is >10% in a sample, and occurring in >10% of all samples.

**Figure 7.**
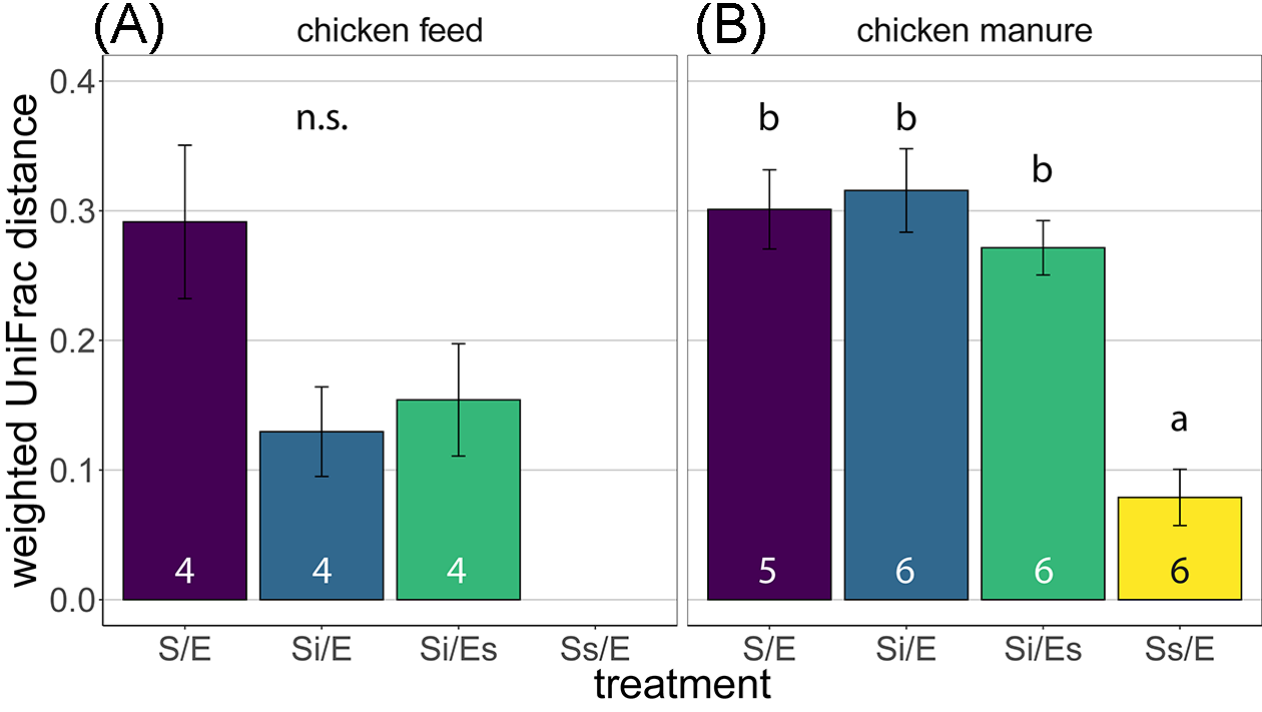
Weighted UniFrac distance between larval and substrate microbiota (mean ± SE): **(A)** chicken feed; **(B)** chicken manure. Treatment codes: S/E = control treatment (untreated substrate and untreated eggs); Si/E = sterilized substrate with inoculum and untreated eggs; Si/Es = sterilized substrate with inoculum and disinfected eggs; Ss/E = sterilized substrate and untreated eggs. Numbers below bars indicate sample sizes (number of containers). Means with different letters are significantly different (α = 0.05; ANOVA with Tukey contrasts). n.s. = not significant.

In chicken feed on day 15, samples of inoculated groups (Si/E, Si/Es) overlapped, suggesting no effect of egg-associated microorganisms on microbiota composition (NMDS; Fig. 5B). However, microbiota of untreated chicken feed differed from the inoculated groups (Si/E, Si/Es) (dbRDA, treatment effect: *P* = 0.002; Table S6, Supporting Information). Eight of the most abundant genera on day 15 were only present in larvae and substrates from untreated chicken feed (S/E; Fig. 6A). Weighted UniFrac distances between larval and substrate microbiota did not differ among treatments (ANOVA, *P* = 0.076; Fig. 7A).

In chicken manure on day 0, substrate microbiota composition of untreated manure (S/E) differed from the inoculated manure (Si/E, Si/Es) (dbRDA, *R*^2^ = 51%, *P* = 0.001; Fig. 5C). There was also a treatment effect on day 15, explaining 63% of total larval and substrate microbiota variation in chicken manure (dbRDA, *P* = 0.001; Fig. 5D; Table S7, Supporting Information). Samples of autoclaved manure (Ss/E) differed from the remaining treatment groups, and untreated manure (S/E) differed from the inoculated manure microbiota (Si/E and Si/Es; Fig. 5D). Twelve of the 22 most abundant genera differed in abundance among larval microbiota of different treatments (Kruskal–Wallis test; Table S8, Supporting Information; Fig. 6B). Larval and substrate microbiota differed in composition in untreated and inoculated manure, but not in autoclaved manure (Fig. 5D). The weighted UniFrac distance between larval and substrate microbiota in autoclaved manure was lower than in the other treatments (ANOVA, *P* < 0.001; Fig. 7B).

## DISCUSSION

This study shows that substrate-associated microorganisms affected larval performance and caused major changes in larval and substrate microbiota, whereas egg-associated microorganisms did not affect performance and only had a minor effect on larval and substrate microbiota.

### Effects of substrate-associated microorganisms on larval performance

BSF larvae performed better on substrates with associated bacteria than on autoclaved substrate without the inoculum (i.e. 10% w/w untreated substrate) (Fig. 2). Larval biomass was lower in the latter treatment in chicken manure compared with all other manure treatments. Moreover, larval development and growth tended to be much retarded in autoclaved chicken feed without inoculum. These effects may have been caused by the differences in bacterial abundance, since initial bacterial abundance in the autoclaved treatments without inoculum was much lower than in the inoculated or untreated manure and chicken feed (Fig. 3). Bacteria serve directly as food for fly larvae and help decompose macronutrients (Gold *et al*. 2018). Improved nutrition is also likely the reason why bacterial inoculation of substrates can lead to increased larval weight (Somroo *et al*. 2019; Mazza *et al*. 2020).

Differences in substrate pH of the manure treatments on day 0 were likely caused by autoclaving, due to the evaporation of ammonia and elimination of bacteria responsible for its production (Erickson *et al*. 2004). The elimination of nitrogen-mineralizing bacteria may also explain why the pH of autoclaved chicken feed on day 15 was so much lower than that of the other chicken feed treatments (Fig. S2, Supporting Information), since bacterial abundance in this substrate remained similar to NTCs in qPCR (Fig. 3).

Larvae fed autoclaved chicken feed with inoculum (Si/E, Si/Es) tended to weigh less than larvae fed untreated chicken feed (Fig. 2B). This difference may be due to the effect of autoclaving on nutritional properties of the feed substrate: complex reactions such as the Maillard reaction, aggregation of proteins and glycation may have reduced protein digestibility and quality (O'Brien, Morrissey and Ames 1989; Nielsen de Almeida 2013). Alternatively, the lower larval weight in the inoculated chicken feed groups may be the result of fiercer competition for food due to the higher larval survival in these groups compared with the untreated chicken feed (Fig. 2A). Larval survival in the untreated chicken feed may have been lower because of a higher moisture content. This is not very likely, however, because moisture content among the chicken feed treatments varied much less than in studies where BSF larval survival was affected by moisture content (Table S1 and Fig. S3, Supporting Information; Fatchurochim, Geden and Axtell 1989; Cammack and Tomberlin 2017; Cheng, Chiu and Lo 2017).

### Effects of substrate-associated microorganisms on larval microbiota

The present study as well as previous studies suggests that especially substrate-associated bacteria influenced the gut microbiota of larvae, and less so the other way around (Bruno *et al*. 2019; Jiang *et al*. 2019; Zhan *et al*. 2020; Schreven *et al*. submitted). The BSF larval gut and substrate exchange large proportions of microbiota over time, with 86% of bacteria in the larval gut samples originating from the substrate and 13% of bacteria in the substrate originating from the larval gut, after larvae were reared on food waste for 10 days (Jiang *et al*. 2019). The difference between larval and substrate microbiota is caused by differences in prevailing environmental conditions, including the selection pressure of the larval digestive and immune systems (Vogel *et al*. 2018; Bonelli *et al*. 2019; Bruno *et al*. 2019; Schreven *et al*. submitted).

Larval and substrate microbiota differed significantly in both untreated and inoculated manure, like previously found (Schreven *et al*. submitted), but did not differ in autoclaved manure (Figs 5 –7). This may have several explanations related to the immune response of BSF larvae. A high bacterial load of a mixture of bacteria, present in the untreated and inoculated manure, can trigger a strong and complex larval immune response (Vogel *et al*. 2018), but the initial bacterial abundance in the autoclaved manure was a thousand times lower, compared with the other treatments (Fig. 3). Second, the bacterial species present in the autoclaved manure could have triggered a different immune response from those present in the other treatments (Zdybicka-Barabas *et al*. 2017). Finally, Erickson *et al*. (2004) suggest that active larval growth is linked to a decrease in *Salmonella* populations in manure, and absence of growth may imply a loss of this effect. Hence, the reduced larval growth we observed in autoclaved manure (Fig. 2B) may have led to reduced suppression of certain bacteria as well, and consequently the higher similarity between larval and substrate microbiota in this treatment (Fig. 7B).

In contrast to what we observed in chicken feed, larvae from autoclaved manure without inoculum and with untreated eggs (Ss/E) harboured a bacterial community as abundant as those of the other treatments after 15 days, and these bacteria may have originated from the eggs. *Bacillus*, *Lysinibacillus* and *Oceanobacillus* dominated larval and substrate microbiota in this treatment (Fig. 6). *Lysinibacillus fusiformis* was previously isolated from the eggs of our BSF colony (Schreven *et al*. unpublished data) and can increase larval weight and survival (Portela Cardenas 2020). This could indicate that in the absence of competition from substrate-associated microorganisms, egg-associated microorganisms can colonize the substrate. Alternatively, since bacterial abundance in the autoclaved manure without inoculum was considerable on day 0 (10^9^ 16S rRNA gene copies g^–1^), at least part of the bacterial DNA may be extracellular DNA from dead bacteria and therefore 16S rRNA gene copy numbers may overestimate the viable bacterial population (Carini *et al*. 2016; Emerson *et al*. 2017).

### The role of egg-associated microorganisms in larval performance and microbiota

Our study suggests that the egg-associated microorganisms were so few compared with the substrate-associated microorganisms that they had no effect on overall microbiota composition or larval performance in both chicken feed and chicken manure (Figs 3–7). They did, however, cause differences in the relative abundance of individual genera and phylogenetic diversity in larvae fed chicken manure (Si/E vs Si/Es; Fig. 4; Table S8, Supporting Information), and in substrate pH in chicken feed (Fig. S2, Supporting Information). Among the most abundant genera, an unassigned genus of Bacillaceae was less abundant in larvae of Si/E than in those of Si/Es manure (Fig. 6; Table S8, Supporting Information). In chicken feed, substrate pH was higher in inoculated diet with disinfected eggs than in inoculated diet with untreated eggs. The higher pH is likely due to increased ammonia production from proteolysis (Erickson *et al*. 2004; Green and Popa 2012). Since there are no differences in larval weight, bacterial abundance or community composition, this suggests that egg-associated microorganisms would suppress the rate of nitrogen mineralization in the chicken feed substrate, through yet unknown mechanisms.

Our findings on the limited role of egg-associated microorganisms in larval performance contrast to previously reported effects of egg-associated or larva-associated bacteria on larval growth (Yu *et al*. 2011; Xiao *et al*. 2018; Mazza *et al*. 2020). A fundamental difference between these studies and ours is that we tested the effect of the total community of microorganisms residing on the untreated eggs, whereas other studies tested single species or mixtures of up to four species of bacteria (Yu *et al*. 2011; Xiao *et al*. 2018; Mazza *et al*. 2020). Moreover, the number of bacteria on the eggs may have been much smaller than applied in the inoculation studies, i.e. 10^8^–10^9^ CFU mL^–1^ inoculum resulting in 10^6^ CFU g^–1^ substrate (Yu *et al*. 2011; Xiao *et al*. 2018; Mazza *et al*. 2020). In our qPCR results, the number of bacterial 16S rRNA gene copies in egg samples was at most 10^8^ copies g^–1^ eggs (Fig. S4, Supporting Information), which would be more diluted still in the substrate. The bacteria that are present on the eggs could still be beneficial to BSF, e.g. during larval hatching from the eggs (Yang *et al*. 2018)—a developmental stage we did not include in our performance study since we used neonate larvae that successfully hatched. Alternatively, Gold *et al*. (2020) suggested a role of larva-associated microorganisms in providing essential nutrients such as vitamins, because they found that sterile BSF larvae failed to grow on autoclaved substrates, whereas non-sterile larvae were able to grow. We may have missed this effect, because we supplemented vitamins to all substrates and did not test the combination of disinfected eggs on autoclaved substrate.

We could not consistently detect and describe the bacterial community present on untreated eggs. Egg samples, untreated or disinfected, showed *C*_q_ values close to or within the range of NTCs. Bacterial densities on BSF eggs may simply be very low and, when extracted from limited starting material (on average 40 mg eggs per sample in our study), too low to be detected by qPCR. In that case, DNA of laboratory and kit contaminants may be present in similar or higher quantities than egg bacterial DNA. Additionally, eukaryotic DNA of the insect may interfere with or be co-amplified by the 16S rRNA gene primers, besides other inhibitors and contaminants extracted with the DNA (Huys *et al*. 2008; Prosdocimi *et al*. 2015). The barcoded PCR of egg samples yielded little product after 30 cycles (<5 ng DNA μL^–1^) and the composition of PCR replicates showed low reproducibility. Zheng *et al*. (2013) successfully sequenced the BSF egg microbiota but used 250 mg eggs. This suggests that with a higher amount of starting material, sequencing of egg-associated bacterial DNA can be successful.

Characterizing the egg-associated microbiota and quantifying its consistency within and among BSF populations over time may provide insights into the flexibility of host–microbe associations in BSF and help explain the variability in members of a core community of BSF larvae across studies (Wynants *et al*. 2019; Khamis *et al*. 2020; Schreven *et al*. submitted). In the present study, *Providencia* was virtually absent, whereas it was strongly associated with larvae regardless of feed substrate in a previous study using eggs of the same BSF colony (Schreven *et al*. submitted). This suggests that besides variability due to host strain, there may be inter-batch variation in egg-associated microbiota. It would be very useful if future research would quantify this variation and investigate its causes.

## CONCLUSION

Our study shows that substrate-associated microorganisms have a larger effect on BSF larval performance and microbiota than egg-associated microorganisms. Substrate-associated microorganisms increased larval biomass in chicken manure, and larval survival and biomass tended to be lower in autoclaved as compared with inoculated chicken feed. Besides, substrate-associated microorganisms increased substrate pH in chicken feed, likely related to increased ammonia production. In chicken manure, substrate-associated microorganisms accounted for major shifts in larval and substrate microbiota: autoclaved manure with larvae from untreated eggs resulted in a high similarity between larval and substrate microbiota, different from the microbiota in the other manure treatments. This may indicate that the larval digestive or immune systems were triggered differently in this treatment compared with the other manure treatments.

Although previous studies showed that egg-associated bacteria can increase larval performance if applied to the substrate in higher concentrations, we found no such effect of the egg-associated microorganisms as present in resident concentrations on the eggs. We also did not record an effect of egg-associated microorganisms on overall microbiota composition. However, their presence resulted in decreased pH in chicken feed and increased phylogenetic diversity of larval microbiota from chicken manure. In conclusion, we found large effects of substrate-associated microorganisms and only minor effects of egg-associated microorganisms, indicating that BSF producers would better focus on manipulation of the former to improve BSF performance and microbiological safety.

## ACKNOWLEDGEMENTS

We thank Hans Smid and Janneke Bloem for their advice in developing the protocols for egg disinfection and further lab work. Ineke Heikamp-De Jong, Steven Aalvink, Merlijn van Gaal and Philippe Puylaert are thanked for support in molecular analyses. We are grateful for the generous advice and help of Ruth Gomez-Exposito and Prokopis Konstanti in sample processing and downstream steps. We thank Pieter Rouweler, André Gidding and Frans van Aggelen for maintaining the insect colony, and André Maassen and Henk Smid of the Unifarm facility (Wageningen University & Research) for autoclaving the substrates. Bart Nijsse and Daan Mertens are thanked for their help in data analysis. We thank Wim van der Putten (NIOO-KNAW), Guido Bosch (Wageningen University) and teachers of the New Frontiers in Microbial Ecology course 2018 (University of Groningen) for suggestions and discussions on the experimental design. Finally, we thank Max Wantulla, Filippo Guerra, Sandeep Sarde, Julia Friman and Yidong Wang for their help during lab work.

## Supplementary Material

fiab054_Supplemental_Files

## References

[bib1] Barragán-Fonseca KB , Dicke M, Van Loon JJA. Nutritional value of the black soldier fly (*Hermetia illucens* L.) and its suitability as animal feed: a review. J Insects Food Feed. 2017;3:105–20.

[bib2] Bates D , Maechler M, Bolker B et al. Fitting linear mixed-effects models using lme4. J Stat Softw. 2015;67:1–48.

[bib3] Benbow ME , Barton PS, Ulyshen MD et al. Necrobiome framework for bridging decomposition ecology of autotrophically and heterotrophically derived organic matter. Ecol Monogr. 2019;89:e01331.

[bib4] Benjamini Y , Hochberg Y. Controlling the false discovery rate: a practical and powerful approach to multiple testing. J R Stat Soc Ser B Stat Methodol. 1995;57:289–300.

[bib6] Bonelli M , Bruno D, Brilli M et al. Black soldier fly larvae adapt to different food substrates through morphological and functional responses of the midgut. Int J Mol Sci. 2020;21:4955. 10.3390/ijms21144955 PMC7404193 32668813

[bib5] Bonelli M , Bruno D, Caccia S et al. Structural and functional characterization of *Hermetia illucens* larval midgut. Front Physiol. 2019;10:204. 30906266 10.3389/fphys.2019.00204 PMC6418021

[bib7] Bosch G , Van Zanten HHE, Zamprogna A et al. Conversion of organic resources by black soldier fly larvae: legislation, efficiency and environmental impact. J Cleaner Prod. 2019;222:355–63.

[bib8] Bruno D , Bonelli M, De Filippis F et al. The intestinal microbiota of *Hermetia illucens* larvae is affected by diet and shows a diverse composition in the different midgut regions. Appl Environ Microbiol. 2019;85:e01864–18. 30504212 10.1128/AEM.01864-18 PMC6328772

[bib9] Buser CC , Newcomb RD, Gaskett AC et al. Niche construction initiates the evolution of mutualistic interactions. Ecol Lett. 2014;17:1257–64. 25041133 10.1111/ele.12331

[bib10] Cammack JA , Tomberlin JK. The impact of diet protein and carbohydrate on select life-history traits of the black soldier fly *Hermetia illucens* (L.) (Diptera: Stratiomyidae). Insects. 2017;8:56. 10.3390/insects8020056 PMC5492070 28561763

[bib11] Carini P , Marsden PJ, Leff JW et al. Relic DNA is abundant in soil and obscures estimates of soil microbial diversity. Nat Microbiol. 2017;2:16242. 10.1038/nmicrobiol.2016.242 27991881

[bib12] Cheng JYK , Chiu SLH, Lo IMC. Effects of moisture content of food waste on residue separation, larval growth and larval survival in black soldier fly bioconversion. Waste Manag. 2017;67:315–23. 28587803 10.1016/j.wasman.2017.05.046

[bib13] Cickova H , Newton GL, Lacy RC et al. The use of fly larvae for organic waste treatment. Waste Manag. 2015;35:68–80. 25453313 10.1016/j.wasman.2014.09.026

[bib14] De Smet J , Wynants E, Cos P et al. Microbial community dynamics during rearing of black soldier fly larvae (*Hermetia illucens*) and impact on exploitation potential. Appl Environ Microbiol. 2018;84:e02722–17. 29475866 10.1128/AEM.02722-17 PMC5930328

[bib15] EFSA . Risk profile related to production and consumption of insects as food and feed. EFSA J. 2015;13:4257.

[bib16] Emerson JB , Adams RI, Roman CMB et al. Schrodinger's microbes: tools for distinguishing the living from the dead in microbial ecosystems. Microbiome. 2017;5:86. 28810907 10.1186/s40168-017-0285-3 PMC5558654

[bib17] Engel P , Moran NA. The gut microbiota of insects: diversity in structure and function. FEMS Microbiol Rev. 2013;37:699–735. 23692388 10.1111/1574-6976.12025

[bib18] Erickson MC , Islam M, Sheppard C et al. Reduction of *Escherichia coli* O157:H7 and *Salmonella enterica* serovar Enteritidis in chicken manure by larvae of the black soldier fly. J Food Prot. 2004;67:685–90. 15083719 10.4315/0362-028x-67.4.685

[bib19] Faith DP . Conservation evaluation and phylogenetic diversity. Biol Conserv. 1992;61:1–10.

[bib20] Fatchurochim S , Geden CJ, Axtell RC. Filth fly (Diptera) oviposition and larval development in poultry manure of various moisture levels. J Entomol Sci. 1989;24:224–31.

[bib22] Gold M , Binggeli M, Kurt F et al. Novel experimental methods for the investigation of *Hermetia illucens* (Diptera: Stratiomyidae) larvae. J Insect Sci. 2020;20:21. 10.1093/jisesa/ieaa057 PMC7320877 32593171

[bib21] Gold M , Tomberlin JK, Diener S et al. Decomposition of biowaste macronutrients, microbes, and chemicals in black soldier fly larval treatment: a review. Waste Manag. 2018;82:302–18. 30509593 10.1016/j.wasman.2018.10.022

[bib23] Green TR , Popa R. Enhanced ammonia content in compost leachate processed by black soldier fly larvae. Appl Biochem Biotechnol. 2012;166:1381–7. 22238016 10.1007/s12010-011-9530-6

[bib24] Hanski I . Nutritional ecology of dung-and carrion-feeding insects. In: Slansky Jr F, Rodriguez JG, (eds). Nutritional Ecology of Insects, Mites, Spiders, and Related Invertebrates. New York: Wiley, 1987, 837–84.

[bib25] Hellemans J , Mortier G, De Paepe A et al. qBase relative quantification framework and software for management and automated analysis of real-time quantitative PCR data. Genome Biol. 2007;8:R19. 17291332 10.1186/gb-2007-8-2-r19 PMC1852402

[bib26] Huys G , Vanhoutte T, Joossens M et al. Coamplification of eukaryotic DNA with 16S rRNA gene-based PCR primers: possible consequences for population fingerprinting of complex microbial communities. Curr Microbiol. 2008;56:553–7. 18301945 10.1007/s00284-008-9122-z

[bib27] Jeon H , Park S, Choi J et al. The intestinal bacterial community in the food waste-reducing larvae of *Hermetia illucens*. Curr Microbiol. 2011;62:1390–9. 21267722 10.1007/s00284-011-9874-8

[bib28] Jiang CL , Jin WZ, Tao XH et al. Black soldier fly larvae (*Hermetia illucens*) strengthen the metabolic function of food waste biodegradation by gut microbiome. Microb Biotechnol. 2019;12:528–43. 30884189 10.1111/1751-7915.13393 PMC6465238

[bib29] Khamis FM , Ombura FLO, Akutse KS et al. Insights in the global genetics and gut microbiome of black soldier fly, *Hermetia illucens*: implications for animal feed safety control. Front Microbiol. 2020;11:1538. 32774330 10.3389/fmicb.2020.01538 PMC7381391

[bib30] Kim E , Park J, Lee S et al. Identification and physiological characters of intestinal bacteria of the black soldier fly, *Hermetia illucens*. Korean J Appl Entomol. 2014;53:15–26.

[bib31] Klammsteiner T , Walter A, Bogataj T et al. The core gut microbiome of black soldier fly (*Hermetia illucens*) larvae raised on low-bioburden diets. Front Microbiol. 2020;11:993. 32508795 10.3389/fmicb.2020.00993 PMC7253588

[bib32] Kruskal JB. Nonmetric multidimensional scaling: a numerical method. Psychometrika. 1964;29:115–29.

[bib33] Lahti L , Shetty SA. Tools for Microbiome Analysis in R. Microbiome Package Version 1.2.1. 2017.

[bib34] Lalander C , Diener S, Magri ME et al. Faecal sludge management with the larvae of the black soldier fly (*Hermetia illucens*): from a hygiene aspect. Sci Total Environ. 2013;458–60:312–8. 10.1016/j.scitotenv.2013.04.033 23669577

[bib35] Lam K , Thu K, Tsang M et al. Bacteria on housefly eggs, *Musca domestica*, suppress fungal growth in chicken manure through nutrient depletion or antifungal metabolites. Naturwissenschaften. 2009;96:1127–32. 19636523 10.1007/s00114-009-0574-1

[bib36] Lee CM , Lee YS, Seo SH et al. Screening and characterization of a novel cellulase gene from the gut microflora of *Hermetia illucens* using metagenomic library. J Microbiol Biotechnol. 2014;24:1196–206. 25022521 10.4014/jmb.1405.05001

[bib37] Lenth R . Emmeans: Estimated Marginal Means, Aka Least-Squares Means. R package version 1.4.5. 2020.

[bib38] Luzopone C , Knight R. UniFrac: a new phylogenetic method for comparing microbial communities. Appl Environ Microbiol. 2005;71:8228–35. 16332807 10.1128/AEM.71.12.8228-8235.2005 PMC1317376

[bib39] Mazza L , Xiao X, Ur Rehman K et al. Management of chicken manure using black soldier fly (Diptera: Stratiomyidae) larvae assisted by companion bacteria. Waste Manag. 2020;102:312–8. 31707320 10.1016/j.wasman.2019.10.055

[bib40] McArdle BH , Anderson MJ. Fitting multivariate models to community data: a comment on distance-based redundancy analysis. Ecology. 2001;82:290–7.

[bib41] McMurdie PJ , Holmes S. phyloseq: an R package for reproducible interactive analysis and graphics of microbiome census data. PLoS One. 2013;8:e61217. 23630581 10.1371/journal.pone.0061217 PMC3632530

[bib42] Nielsen de Almeida F . Effects of the Maillard Reactions on Chemical Composition and Amino Acid Digestibility of Feed Ingredients and on Pig Growth Performance. Urbana, IL: University of Illinois, 2013.

[bib43] O'Brien J , Morrissey PA, Ames JM. Nutritional and toxicological aspects of the Maillard browning reaction in foods. Crit Rev Food Sci Nutr. 1989;28:211–48. 2669832 10.1080/10408398909527499

[bib44] Oksanen J , Blanchet FG, Friendly M et al. vegan: Community Ecology Package. R package version 2.5-6. 2019.

[bib45] Pastor B , Velasquez Y, Gobbi P et al. Conversion of organic wastes into fly larval biomass: bottlenecks and challenges. J Insects Food Feed. 2015;1:179–93.

[bib46] Pinheiro J , Bates D, DebRoy S et al. Nlme: Linear and Nonlinear Mixed Effects Models. R Package Version 3.1-137. 2018.

[bib47] Poncheewin W , Hermes GDA, van Dam JCJ et al. NG-Tax 2.0: a semantic framework for high-throughput amplicon analysis. Front Genet. 2019;10:1366. 32117417 10.3389/fgene.2019.01366 PMC6989550

[bib48] Portela Cardenas MD . Testing the Effect of Two Bacterial Species on Survival and Growth of Black Soldier Fly Larvae. B.Sc. Thesis, Wageningen, The Netherlands: Wageningen University & Research, 2020.

[bib49] Prosdocimi EM , Mapelli F, Gonella E et al. Microbial ecology-based methods to characterize the bacterial communities of non-model insects. J Microbiol Methods. 2015;119:110–25. 26476138 10.1016/j.mimet.2015.10.010

[bib50] Quast C , Pruesse E, Yilmaz P et al. The SILVA ribosomal RNA gene database project: improved data processing and web-based tools. Nucleic Acids Res. 2013;41:D590–6. 23193283 10.1093/nar/gks1219 PMC3531112

[bib52] Ramiro-Garcia J , Hermes GDA, Giatsis C et al. NG-Tax, a highly accurate and validated pipeline for analysis of 16S rRNA amplicons from complex biomes. F1000Res. 2016;5:1791. 30918626 10.12688/f1000research.9227.1 PMC6419982

[bib51] R Core Team . R: A Language and Environment for Statistical Computing. Version 3.5.0. Vienna, Austria: R Foundation for Statistical Computing, 2018.

[bib53] Sakamoto Y , Ishiguro M, Kitagawa G. Akaike Information Criterion Statistics. Dordrecht, The Netherlands: D. Reidel Publishing Company, 1986.

[bib54] Salonen A , Nikkila J, Jalanka-Tuovinen J et al. Comparative analysis of fecal DNA extraction methods with phylogenetic microarray: effective recovery of bacterial and archaeal DNA using mechanical cell lysis. J Microbiol Methods. 2010;81:127–34. 20171997 10.1016/j.mimet.2010.02.007

[bib55] Salter SJ , Cox MJ, Turek EM et al. Reagent and laboratory contamination can critically impact sequence-based microbiome analyses. BMC Biol. 2014;12:87. 25387460 10.1186/s12915-014-0087-z PMC4228153

[bib56] Schreven SJJ , de Vries H, Hermes GDA et al. Substrate-Dependent Impact of Black Soldier Fly Larvae on Bacterial Community Composition in Substrate and Larval Gut. Submitted.

[bib57] Skaro M . Influence of Addition of Bacterial Cultures Into the Rearing Substrate on Microflora of Black Soldier Fly Larvae. M.Sc. Thesis, Zagreb, Croatia: University of Zagreb, 2018.

[bib58] Somroo AA , Ur Rehman K, Zheng L et al. Influence of *Lactobacillus buchneri* on soybean curd residue co-conversion by black soldier fly larvae (*Hermetia illucens*) for food and feedstock production. Waste Manag. 2019;86:114–22. 30902235 10.1016/j.wasman.2019.01.022

[bib59] Stamps JA , Yang LH, Morales VM et al. *Drosophila* regulate yeast density and increase yeast community similarity in a natural substrate. PLoS One. 2012;7:e42238. 22860093 10.1371/journal.pone.0042238 PMC3409142

[bib60] Thompson CR , Brogan RS, Scheifele LZ et al. Bacterial interactions with necrophagous flies. Ann Entomol Soc Am. 2013;106:799–809.

[bib61] Van Lingen HJ , Edwards JE, Vaidya JD et al. Diurnal dynamics of gaseous and dissolved metabolites and microbiota composition in the bovine rumen. Front Microbiol. 2017;8:425. 28367142 10.3389/fmicb.2017.00425 PMC5355475

[bib62] Vogel H , Muller A, Heckel DG et al. Nutritional immunology: diversification and diet-dependent expression of antimicrobial peptides in the black soldier fly *Hermetia illucens*. Dev Comp Immunol. 2018;78:141–8. 28966127 10.1016/j.dci.2017.09.008

[bib63] Wang YS , Shelomi M. Review of black soldier fly (*Hermetia illucens*) as animal feed and human food. Foods. 2017;6:91. 10.3390/foods6100091 PMC5664030 29057841

[bib64] Wynants E , Frooninckx L, Crauwels S et al. Assessing the microbiota of black soldier fly larvae (*Hermetia illucens*) reared on organic waste streams on four different locations at laboratory and large scale. Microb Ecol. 2019;77:913–30. 30430196 10.1007/s00248-018-1286-x

[bib65] Xiao X , Mazza L, Yu Y et al. Efficient co-conversion process of chicken manure into protein feed and organic fertilizer by *Hermetia illucens* L. (Diptera: Stratiomyidae) larvae and functional bacteria. J Environ Manage. 2018;217:668–76. 29654970 10.1016/j.jenvman.2018.03.122

[bib66] Yang QQ , Wang H, Liu X et al. The effect of the egg associated bacteria on the hatching of *Hermetia illucens* eggs. J Insects Food Feed. 2018;4:82.

[bib67] Yu G , Cheng P, Chen Y et al. Inoculating poultry manure with companion bacteria influences growth and development of black soldier fly (Diptera: Stratiomyidae) larvae. Environ Entomol. 2011;40:30–5. 22182608 10.1603/EN10126

[bib68] Yu GH , Niu CY, He GB et al. Isolation and identification of bacteria producing enzymes from gut and skin of black soldier fly (*Hermetia illucens*) larvae. Chin Bull Entomol. 2010;47:889–94.

[bib69] Zdybicka-Barabas A , Bulak P, Polakowski C et al. Immune response in the larvae of the black soldier fly *Hermetia illucens*. Invertebr Surviv J. 2017;14:9–17.

[bib70] Zhan S , Fang G, Cai M et al. Genomic landscape and genetic manipulation of the black soldier fly *Hermetia illucens*, a natural waste recycler. Cell Res. 2020;30:50–60. 31767972 10.1038/s41422-019-0252-6 PMC6951338

[bib72] Zheng L , Crippen TL, Singh B et al. A survey of bacterial diversity from successive life stages of black soldier fly (Diptera: Stratiomyidae) by using 16S rDNA pyrosequencing. J Med Entomol. 2013;50:647–58. 23802462 10.1603/me12199

[bib71] Zheng L , Hou Y, Li W et al. Biodiesel production from rice straw and restaurant waste employing black soldier fly assisted by microbes. Energy. 2012;47:225–9.

