# Supplementary material for: Relative contributions of egg-associated and substrate-associated microorganisms to black soldier fly larval performance and microbiota
Source: FEMS Microbiol Ecol. 2021 Mar 30;97(5):fiab054. doi: 10.1093/femsec/fiab054 (PMC8044291; doi:10.1093/femsec/fiab054)
Supplement: fiab054_Supplemental_Files [file fiab054_supplemental_files.zip › FEMS_Schreven_et_al_Supplementary_file_captions_revised_Acc.docx]

**Supplementary files captions, Schreven et al. “Relative contributions of egg-associated and substrate-associated microorganisms to black soldier fly larval performance and microbiota”:**

Supplementary Tables (1 file, 9 tables, 1 table per page):

Supplementary Table 1. Amount of feed (g dry matter) and moisture content (% of fresh matter) per feed substrate and treatment on day 0, mean ± SE. Treatment codes: S/E = control treatment (untreated substrate and untreated eggs), Si/E = sterilized substrate with inoculum and untreated eggs, Si/Es = sterilized substrate with inoculum and disinfected eggs, Ss/E = sterilized substrate and untreated eggs. Means that share no letters are significantly different (per parameter and per feed substrate, LMM with Tukey contrasts, α = 0.05).

Supplementary Table 2. Analysis of deviance table for substrate pH of chicken feed, GLMM regression model.

Supplementary Table 3. Analysis of deviance table for substrate pH of chicken manure, GLMM regression model.

Supplementary Table 4. Total relative abundance (of all reads in dataset) of ASVs identified as contaminants, grouped per genus and ordered by relative abundance. The thirty most abundant genera are displayed, the rest is summed under “Other”. Contaminant identification based on assessment of correlation plots between ASV relative abundance and DNA concentration of samples.

Supplementary Table 5. Spearman rank correlations (mean ± SD) between technical replicates of samples. Treatment codes: S/E = control treatment (untreated substrate and untreated eggs), Si/Es = sterilized substrate with inoculum and disinfected eggs.

Supplementary Table 6. Output of permutational multivariate ANOVA of weighted UniFrac dbRDA of chicken feed on day 15. 999 permutations, stratified for container ID. R^2^ = 54%.

Supplementary Table 7. Output of permutational multivariate ANOVA of weighted UniFrac dbRDA of chicken manure on day 15. 999 permutations, stratified for container ID. R^2^ = 75%.

Supplementary Table 8. Genera with differential relative abundance among larval microbiota of different treatments of chicken manure on day 15. Treatment codes: S/E = control treatment (untreated substrate and untreated eggs), Si/E = sterilized substrate with inoculum and untreated eggs, Si/Es = sterilized substrate with inoculum and disinfected eggs, Ss/E = sterilized substrate and untreated eggs. Kruskal-Wallis test and posthoc Wilcoxon tests, with FDR-corrected P-values. Treatment groups without shared letters have significantly different medians (α = 0.05), letter a is lowest median.

Supplementary Table 9. Larval performance and substrate pH data. Treatment codes: S/E = control treatment (untreated substrate and untreated eggs), Si/E = sterilized substrate with inoculum and untreated eggs, Si/Es = sterilized substrate with inoculum and disinfected eggs, Ss/E = sterilized substrate and untreated eggs. Larval performance (survival rate, % prepupae, individual larval weight, and total larval biomass) and substrate moisture content were determined on day 15, except for chicken feed treatment Ss/E (on day 22).

Supplementary Figures (5 files, each figure separately):

Supplementary Figure 1. Percentage of prepupae on day 15, in chicken feed (left panel) and chicken manure (right). Treatment codes: S/E = control treatment (untreated substrate and untreated eggs), Si/E = sterilized substrate with inoculum and untreated eggs, Si/Es = sterilized substrate with inoculum and disinfected eggs, Ss/E = sterilized substrate and untreated eggs. Numbers in bars indicate sample sizes (number of containers). Data for chicken feed Ss/E are from day 22. N.s. = not significant (Kruskal-Wallis test, α = 0.05).

Supplementary Figure 2. Substrate pH (mean ± SE) of A) chicken feed and B) chicken manure over time. Treatment codes: S/E = control treatment (untreated substrate and untreated eggs), Si/E = sterilized substrate with inoculum and untreated eggs, Si/Es = sterilized substrate with inoculum and disinfected eggs, Ss/E = sterilized substrate and untreated eggs. Numbers in bars indicate sample sizes (number of containers). Means without shared letters are significantly different (α = 0.05; GLMM per feed substrate; posthoc comparisons with Tukey-corrected P-values).

Supplementary Figure 3. Boxplots of substrate moisture content at time of harvest (% fresh matter). Treatment codes: S/E = control treatment (untreated substrate and untreated eggs), Si/E = sterilized substrate with inoculum and untreated eggs, Si/Es = sterilized substrate with inoculum and disinfected eggs, Ss/E = sterilized substrate and untreated eggs. All treatments were harvested on day 15, except chicken feed Ss/E which was harvested on day 22 and therefore excluded from statistics. Numbers below bars indicate sample sizes. Medians with no shared letters are significantly different (P < 0.05, Kruskal-Wallis test with Wilcoxon posthoc comparisons and FDR-corrected P-values).

Supplementary Figure 4. Boxplots of log_10_ transformed 16S rRNA gene copies per g fresh matter sample material, in egg samples. Samples that scored similar to no-template controls were imputed in the analysis as log_10_(1) = 0, meaning that 16S rRNA gene copy numbers were below the detection threshold. Untreated and disinfected eggs did not differ in 16S rRNA gene abundance (Wilcoxon, P = 0.289). Numbers in boxplots indicate sample sizes (number of batches).

Supplementary Figure 5. Relative abundance (mean – SE) of the five most abundant bacterial phyla in substrate and larvae samples from different treatments in both feed substrates on day 0 and 15. Treatment codes: S/E = control treatment (untreated substrate and untreated eggs), Si/E = sterilized substrate with inoculum and untreated eggs, Si/Es = sterilized substrate with inoculum and disinfected eggs, Ss/E = sterilized substrate and untreated eggs.
