# Supplementary material for: Relative contributions of egg-associated and substrate-associated microorganisms to black soldier fly larval performance and microbiota
Source: FEMS Microbiol Ecol. 2021 Mar 30;97(5):fiab054. doi: 10.1093/femsec/fiab054 (PMC8044291; doi:10.1093/femsec/fiab054)
Supplement: fiab054_Supplemental_Files [file fiab054_supplemental_files.zip › FEMS_Schreven_et_al_Supplementary_Tables_revised_Acc.docx]

Supplementary Tables, Schreven et al. “Relative contributions of egg-associated and substrate-associated microorganisms to black soldier fly larval performance and microbiota”:

**Supplementary Table 1. Amount of feed (g dry matter) and moisture content (% of fresh matter) per feed substrate and treatment on day 0, mean ± SE.** Treatment codes: S/E = control treatment (untreated substrate and untreated eggs), Si/E = sterilized substrate with inoculum and untreated eggs, Si/Es = sterilized substrate with inoculum and disinfected eggs, Ss/E = sterilized substrate and untreated eggs. Means that share no letters are significantly different (per parameter and per feed substrate, LMM with Tukey contrasts, α = 0.05).

| **Feed substrate** | **Treatment** | **Amount of feed (g DM)** | **Moisture content (% fresh matter)** |
| --- | --- | --- | --- |
| chicken feed | S/E | 18.1 ± 0.3a | 71.3 ± 0.4d |
| chicken feed | Si/E | 19.9 ± 0.3b | 68.4 ± 0.4b |
| chicken feed | Si/Es | 20.0 ± 0.3bc | 69.2 ± 0.4c |
| chicken feed | Ss/E | 20.1 ± 0.3c | 68.1 ± 0.4a |
| chicken manure | S/E | 20.6 ± 0.4a | 67.1 ± 0.5b |
| chicken manure | Si/E | 22.1 ± 0.4b | 65.9 ± 0.5a |
| chicken manure | Si/Es | 21.9 ± 0.4b | 65.8 ± 0.5a |
| chicken manure | Ss/E | 22.0 ± 0.4b | 65.9 ± 0.5a |

**Supplementary Table 2. Analysis of deviance table for substrate pH of chicken feed, GLMM regression model.**

|  | Chisq | Df | P |
| --- | --- | --- | --- |
| Treatment | 60.20 | 3 | < 0.001 |
| Timepoint | 609.56 | 1 | < 0.001 |
| Treatment x Timepoint | 75.47 | 3 | < 0.001 |

**Supplementary Table 3. Analysis of deviance table for substrate pH of chicken manure, GLMM regression model.**

|  | Chisq | Df | P |
| --- | --- | --- | --- |
| Treatment | 9.65 | 3 | 0.022 |
| Timepoint | 73.32 | 1 | < 0.001 |
| Treatment x Timepoint | 13.32 | 3 | 0.004 |

**Supplementary Table 4. Total relative abundance (of all reads in dataset) of ASVs identified as contaminants, grouped per genus and ordered by relative abundance.** The thirty most abundant genera are displayed, the rest is summed under “Other”. Contaminant identification based on assessment of correlation plots between ASV relative abundance and DNA concentration of samples.

| **Genus** | **number of ASVs** | **number of reads** | **% of total reads** |
| --- | --- | --- | --- |
| *Ralstonia* | 62 | 494163 | 1.5469% |
| Unassigned taxon | 26 | 80241 | 0.2512% |
| *Clostridium_sensu_stricto_1* | 7 | 39161 | 0.1226% |
| Peptostreptococcaceae (unassigned genus) | 7 | 30281 | 0.0948% |
| *Fusobacterium* | 3 | 30187 | 0.0945% |
| *Cupriavidus* | 5 | 23194 | 0.0726% |
| *Turicibacter* | 1 | 11178 | 0.0350% |
| *Syntrophococcus* | 5 | 9545 | 0.0299% |
| *Candidatus_Nucleicultrix* | 1 | 9320 | 0.0292% |
| *Lactobacillus* | 6 | 6234 | 0.0195% |
| *Subdoligranulum* | 6 | 4987 | 0.0156% |
| Burkholderiaceae (unassigned genus) | 1 | 4737 | 0.0148% |
| *Achromobacter* | 1 | 3259 | 0.0102% |
| *Stenotrophomonas* | 1 | 3083 | 0.0097% |
| *Sphingomonas* | 4 | 2893 | 0.0091% |
| *Shewanella* | 2 | 2740 | 0.0086% |
| *Desulfovibrio* | 1 | 2594 | 0.0081% |
| *Shuttleworthia* | 1 | 2321 | 0.0073% |
| *Aquabacterium* | 1 | 1954 | 0.0061% |
| *Staphylococcus* | 3 | 1713 | 0.0054% |
| *Holdemanella* | 1 | 1522 | 0.0048% |
| *Collinsella* | 1 | 1478 | 0.0046% |
| *Blautia* | 3 | 1442 | 0.0045% |
| *Catenisphaera* | 1 | 1411 | 0.0044% |
| *Solobacterium* | 2 | 1390 | 0.0044% |
| *Catenibacterium* | 1 | 1372 | 0.0043% |
| *Delftia* | 1 | 1115 | 0.0035% |
| *Faecalibacterium* | 1 | 950 | 0.0030% |
| *Methylobacterium* | 2 | 947 | 0.0030% |
| Lactobacillaceae (unassigned genus) | 2 | 896 | 0.0028% |
| Other | 29 | 8440 | 0.0264% |
| **Total** | **188** | **784748** | **2.4565%** |

**Supplementary Table 5. Spearman rank correlations (mean ± SD) between technical replicates of samples.** Treatment codes: S/E = control treatment (untreated substrate and untreated eggs), Si/Es = sterilized substrate with inoculum and disinfected eggs.

| **Replication** | **Sample ID** | **Sample type** | **Feed substrate** | **Treatment** | **Timepoint** | **N replicates** | **Spearman r** |
| --- | --- | --- | --- | --- | --- | --- | --- |
| DNA extraction | 15.K | substrate | chicken feed | S/E | 0 | 4 | 1.000 ± 0 |
| DNA extraction | 15.N | larvae | chicken feed | S/E | 15 | 4 | 0.968 ± 0.035 |
| DNA extraction | 18.K | substrate | chicken manure | Si/Es | 0 | 4 | 0.828 ± 0.104 |
| DNA extraction | 32.K | substrate | chicken manure | S/E | 0 | 4 | 0.940 ± 0.007 |
| DNA extraction | 30.N | larvae | chicken manure | S/E | 15 | 4 | 0.958 ± 0.008 |
| PCR | 16.K | substrate | chicken feed | S/E | 0 | 2 | 1.000 |
| PCR | 15.N | larvae | chicken feed | S/E | 15 | 2 | 1.000 |
| PCR | 18.N | larvae | chicken manure | Si/Es | 15 | 2 | 0.972 |
| PCR | 33.M | substrate | chicken manure | Si/Es | 15 | 2 | 0.954 |

**Supplementary Table 6. Output of permutational multivariate ANOVA of weighted UniFrac dbRDA of chicken feed on day 15. 999 permutations, stratified for container ID. R^2^ = 54%.**

|  | df | SSq | F | P | % explained |
| --- | --- | --- | --- | --- | --- |
| Treatment | 2 | 0.509 | 8.103 | 0.002 | 41.0% |
| Sample type | 1 | 0.048 | 1.532 | 0.017 | 3.9% |
| Treatment x Sample type | 2 | 0.119 | 1.886 | 0.002 | 9.5% |
| Residual | 18 | 0.566 |  |  |  |

**Supplementary Table 7. Output of permutational multivariate ANOVA of weighted UniFrac dbRDA of chicken manure on day 15. 999 permutations, stratified for container ID. R^2^ = 75%.**

|  | df | SSq | F | P | % explained |
| --- | --- | --- | --- | --- | --- |
| Treatment | 3 | 2.665 | 32.350 | 0.001 | 63.3% |
| Sample type | 1 | 0.325 | 11.837 | 0.001 | 7.7% |
| Treatment x Sample type | 3 | 0.179 | 2.167 | 0.001 | 4.2% |
| Residual | 38 | 1.043 |  |  |  |

**Supplementary Table 8. Genera with differential relative abundance among larval microbiota of different treatments of chicken manure on day 15.** Treatment codes: S/E = control treatment (untreated substrate and untreated eggs), Si/E = sterilized substrate with inoculum and untreated eggs, Si/Es = sterilized substrate with inoculum and disinfected eggs, Ss/E = sterilized substrate and untreated eggs. Kruskal-Wallis test and posthoc Wilcoxon tests, with FDR-corrected P-values. Treatment groups without shared letters have significantly different medians (α = 0.05), letter a is lowest median.

| **Genus** | **P** | **Multiple comparisons** | | | |
| --- | --- | --- | --- | --- | --- |
|  |  | S/E | Si/E | Si/Es | Ss/E |
| *Amphibacillus* | 0.010 | b | b | b | a |
| Bacillaceae (unassigned) | 0.004 | b | b | c | a |
| Clostridia order MBA03 (uncultured) | 0.021 | b | ab | b | a |
| *Enteractinococcus* | 0.007 | b | b | b | a |
| *Glutamicibacter* | 0.021 | b | ab | ab | a |
| *Gottschalkia* | 0.004 | b | c | c | a |
| *Planomicrobium* | 0.029 | ab | b | b | a |
| *Proteiniphilum* | 0.007 | b | b | b | a |
| *Pseudogracilibacillus* | 0.006 | b | b | b | a |
| Ruminococcaceae (unassigned) | 0.007 | b | b | b | a |
| Sphingobacteriaceae (uncultured) | 0.007 | a | b | b | a |
| *Thiopseudomonas* | 0.004 | * |  |  |  |

* only present in S/E.

**Supplementary Table 9. Larval performance and substrate pH data.** Treatment codes: S/E = control treatment (untreated substrate and untreated eggs), Si/E = sterilized substrate with inoculum and untreated eggs, Si/Es = sterilized substrate with inoculum and disinfected eggs, Ss/E = sterilized substrate and untreated eggs. Larval performance (survival rate, % prepupae, individual larval weight, and total larval biomass) and substrate moisture content were determined on day 15, except for chicken feed treatment Ss/E (on day 22).

| ContainerID | Feed substrate | Treatment | pH substrate on day 0 | pH substrate on day 15 | Survival rate (%) | Prepupae (% of larvae) | Individual larval weight (g dry matter) | total larval biomass (g dry matter) | substrate moisture content on day 15 (% fresh matter) |
| --- | --- | --- | --- | --- | --- | --- | --- | --- | --- |
| 1 | chicken feed | Si/Es | 5.45 | 8.16 | 78.0 | 0.0 | 0.019 | 1.498 | 80.2 |
| 2 | chicken feed | Si/Es | 5.54 | 8.10 | 96.9 | 0.0 | 0.046 | 4.324 | 77.2 |
| 3 | chicken feed | Si/Es | 5.71 | 8.24 | 84.0 | 0.0 | 0.016 | 2.041 | 81.2 |
| 4 | chicken feed | Si/Es | 5.79 | 8.46 | 85.4 | 0.0 | 0.012 | 1.610 | 81.5 |
| 5 | chicken feed | Ss/E | 5.47 | 5.44 | 36.2 | 0.0 | 0.002 | 0.065 | 62.5 |
| 6 | chicken feed | Ss/E | 5.50 | 7.20 | 47.9 | 0.0 | 0.003 | 0.135 | 74.7 |
| 7 | chicken feed | Ss/E | 5.59 | 6.95 | 69.1 | 0.0 | 0.019 | 1.203 | 76.1 |
| 8 | chicken feed | Ss/E | 5.62 | 5.78 | 74.5 | 0.0 | 0.055 | 3.878 | 70.5 |
| 9 | chicken feed | Si/E | 5.58 | 8.03 | 84.0 | 0.0 | 0.019 | 1.562 | 79.5 |
| 10 | chicken feed | Si/E | 5.56 | 7.91 | 81.0 | 0.0 | 0.019 | 1.531 | 78.3 |
| 11 | chicken feed | Si/E | 5.73 | 6.75 | 85.0 | 4.7 | 0.033 | 2.831 | 79.3 |
| 12 | chicken feed | Si/E | 5.78 | 7.41 | 85.0 | 2.4 | 0.024 | 2.066 | 80.6 |
| 13 | chicken feed | S/E | 5.62 | 7.28 | 39.0 | 5.1 | 0.059 | 2.297 | 82.1 |
| 14 | chicken feed | S/E | 5.69 | 7.82 | 59.0 | 0.0 | 0.046 | 2.702 | 83.2 |
| 15 | chicken feed | S/E | 5.68 | 8.05 | 51.0 | 66.7 | 0.081 | 4.141 | 84.0 |
| 16 | chicken feed | S/E | 5.63 | 8.49 | 56.0 | 51.8 | 0.068 | 3.808 | 84.3 |
| 17 | chicken manure | Si/Es | 8.72 | 9.14 | 94.6 | 0.0 | 0.007 | 0.945 | 68.0 |
| 18 | chicken manure | Si/Es | 6.67 | 9.29 | 68.3 | 1.1 | 0.007 | 0.675 | 65.9 |
| 19 | chicken manure | Si/Es | 7.84 | 9.23 | 76.0 | 0.0 | 0.012 | 0.924 | 69.4 |
| 20 | chicken manure | Si/Es | 7.77 | 9.27 | 80.2 | 0.0 | 0.011 | 0.891 | 68.6 |
| 21 | chicken manure | Ss/E | 7.39 | 8.75 | 83.0 | 0.0 | 0.006 | 0.531 | 60.2 |
| 22 | chicken manure | Ss/E | 7.39 | 9.02 | 77.0 | 0.0 | 0.007 | 0.547 | 63.0 |
| 23 | chicken manure | Ss/E | 7.46 | 9.05 | 85.0 | 0.0 | 0.007 | 0.595 | 63.7 |
| 24 | chicken manure | Ss/E | 7.65 | 8.99 | 89.0 | 0.0 | 0.005 | 0.481 | 65.0 |
| 25 | chicken manure | Si/E | 8.83 | 9.34 | 85.0 | 0.0 | 0.008 | 0.714 | 69.7 |
| 26 | chicken manure | Si/E | 6.16 | 9.26 | 76.0 | 0.0 | 0.012 | 0.942 | 68.0 |
| 27 | chicken manure | Si/E | 7.55 | 9.29 | 88.0 | 0.0 | 0.008 | 0.739 | 70.7 |
| 28 | chicken manure | Si/E | 7.75 | 9.22 | 83.0 | 0.0 | 0.009 | 0.739 | 68.1 |
| 29 | chicken manure | S/E | 8.97 | 9.10 | 90.0 | 1.1 | 0.010 | 0.855 | 67.3 |
| 30 | chicken manure | S/E | 9.07 | 9.18 | 84.0 | 0.0 | 0.011 | 0.890 | 66.7 |
| 31 | chicken manure | S/E | 8.52 | 9.26 | 67.0 | 0.0 | 0.013 | 0.884 | 70.7 |
| 32 | chicken manure | S/E | 8.42 | 8.95 | 82.0 | 0.0 | 0.014 | 1.148 | 67.7 |
| 33 | chicken manure | Si/Es | 8.62 | 9.32 | 46.3 | 0.0 | 0.015 | 0.664 | 67.0 |
| 34 | chicken manure | Si/Es | 8.68 | 9.34 | 62.8 | 0.0 | 0.011 | 0.673 | 65.4 |
| 35 | chicken manure | Ss/E | 7.73 | 9.00 | 100.0 | 0.0 | 0.005 | 0.530 | 65.1 |
| 36 | chicken manure | Ss/E | 7.49 | 9.01 | 95.0 | 0.0 | 0.005 | 0.456 | 64.5 |
| 37 | chicken manure | Si/E | 8.59 | 9.29 | 63.0 | 0.0 | 0.011 | 0.712 | 67.3 |
| 38 | chicken manure | Si/E | 8.81 | 9.20 | 79.0 | 0.0 | 0.011 | 0.861 | 65.3 |
| 39 | chicken manure | S/E | 8.79 | 9.17 | 70.0 | 0.0 | 0.010 | 0.693 | 72.3 |
| 40 | chicken manure | S/E | 8.49 | 8.93 | 80.0 | 1.3 | 0.013 | 1.048 | 65.7 |
