# Supplementary figures and images for: Relative contributions of egg-associated and substrate-associated microorganisms to black soldier fly larval performance and microbiota
Source: FEMS Microbiol Ecol. 2021 Mar 30;97(5):fiab054. doi: 10.1093/femsec/fiab054 (PMC8044291; doi:10.1093/femsec/fiab054)

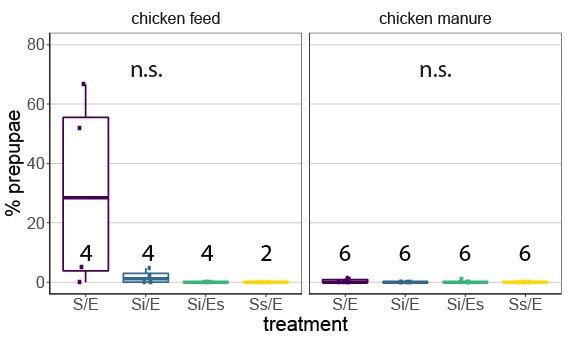

Supplement: fiab054_Supplemental_Files [file fiab054_supplemental_files.zip › Supplementary_Fig_1.jpg]

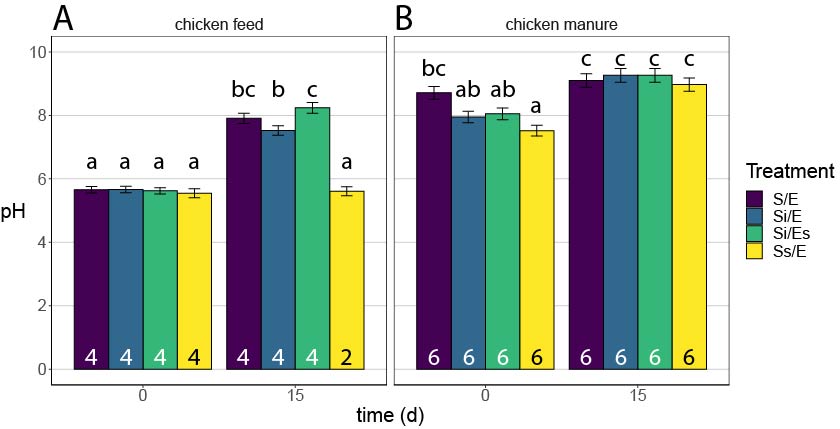

Supplement: fiab054_Supplemental_Files [file fiab054_supplemental_files.zip › Supplementary_Fig_2.jpg]

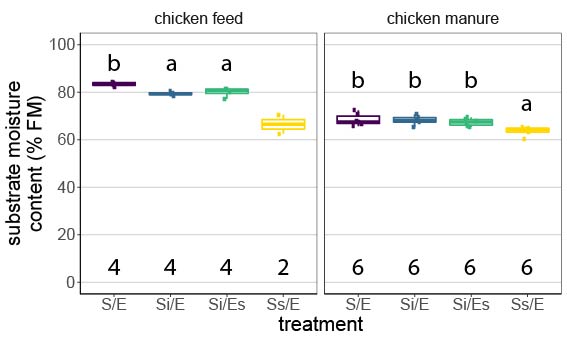

Supplement: fiab054_Supplemental_Files [file fiab054_supplemental_files.zip › Supplementary_Fig_3.jpg]

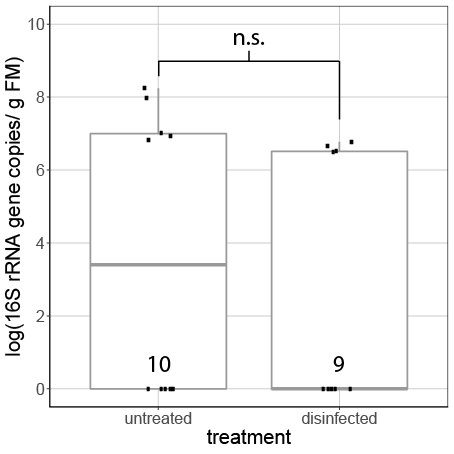

Supplement: fiab054_Supplemental_Files [file fiab054_supplemental_files.zip › Supplementary_Fig_4_revised.jpg]

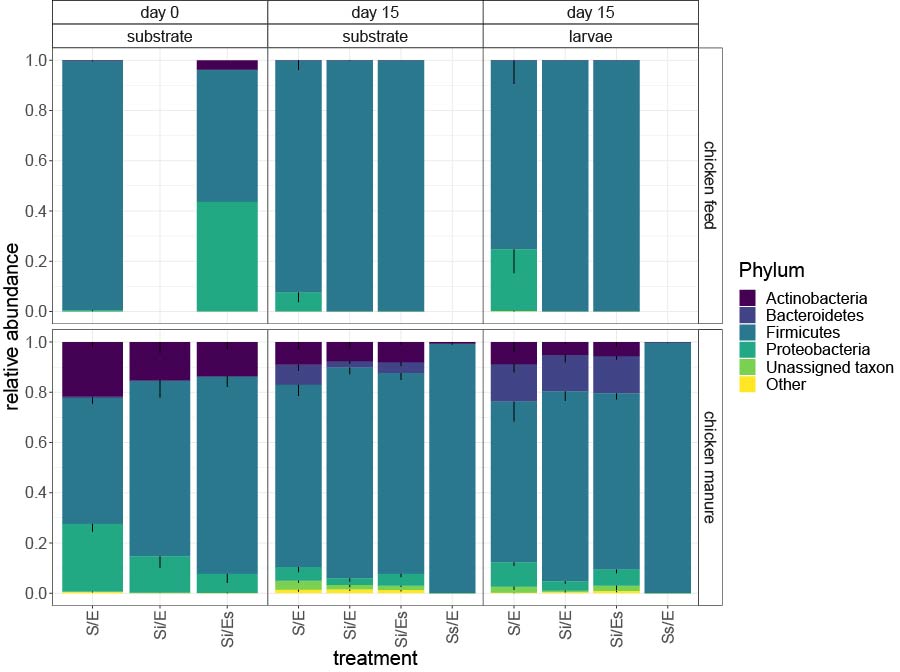

Supplement: fiab054_Supplemental_Files [file fiab054_supplemental_files.zip › Supplementary_Fig_5.jpg]
